# Bulk heterojunction morphology of polymer:fullerene blends revealed by ultrafast spectroscopy

**DOI:** 10.1038/srep36236

**Published:** 2016-11-08

**Authors:** Almis Serbenta, Oleg V. Kozlov, Giuseppe Portale, Paul H. M. van Loosdrecht, Maxim S. Pshenichnikov

**Affiliations:** 1 Zernike Institute for Advanced Materials, University of Groningen, Groningen, the Netherlands; 2 International Laser Center and Faculty of Physics, Moscow State University, Russian Federation

## Abstract

Morphology of organic photovoltaic bulk heterojunctions (BHJs) – a nanoscale texture of the donor and acceptor phases – is one of the key factors influencing efficiency of organic solar cells. Detailed knowledge of the morphology is hampered by the fact that it is notoriously difficult to investigate by microscopic methods. Here we all-optically track the exciton harvesting dynamics in the fullerene acceptor phase from which subdivision of the fullerene domain sizes into the mixed phase (2–15 nm) and large (>50 nm) domains is readily obtained via the Monte-Carlo simulations. These results were independently confirmed by a combination of X-ray scattering, electron and atomic-force microscopies, and time-resolved photoluminescence spectroscopy. In the large domains, the excitons are lost due to the high energy disorder while in the ordered materials the excitons are harvested with high efficiency even from the domains as large as 100 nm due to the absence of low-energy traps. Therefore, optimizing of blend nanomorphology together with increasing the material order are deemed as winning strategies in the exciton harvesting optimization.

Organic solar cells (OSCs) have steadily overcome the important threshold of 10% efficiency^1^,^2^, which makes them a promising alternative to conventional silicon-based solar cells, in particular for niche applications. A typical OSC relies on the ability of strongly-bound photogenerated Frenkel excitons to diffuse to the interface between donor- and acceptor-type materials which provide a driving energy for the exciton splitting^3^. Due to the limited exciton diffusion length in organic materials (~10 nm)^3^,^4^,^5^, there is a compelled compromise between the photon harvesting efficiency that requires 100-nm thick absorber layers, and exciton harvesting efficiency that necessitates relatively short exciton diffusion distances. Different approaches were utilized to maximize the exciton and photon harvesting efficiencies in organic devices, e.g. employing multi-layered structures with light-harvesting and charge-transport layers (*p-i-n* devices)^6^ or creating interpenetrated polymer network^7^. Eventually, this paradox has been triumphantly resolved by utilizing the bulk heterojunction (BHJ) donor-acceptor architecture^8^, which is the most widely used active layer for the OSCs nowadays.

BHJ is basically a nano-textured mixture of organic donor and acceptor materials. To maximize the efficiency of the OSC, the BHJ has to fulfill a number of requirements: (i). sufficient thickness (~100 nm) for efficient photon harvesting, (ii). Fine intermixing (~10 nm) of the ingredients to ensure close-to-unity exciton harvesting, and (iii). intercalated pathways to deliver the charges to the electrodes. The particular nanostructure of the BHJ, the BHJ morphology, is a crucial factor which decisively influences the efficiency of OSCs, and as such it has to be carefully optimized and characterized. So far, there is no solid theory to predict nor a systematic method to control the self-organization of BHJs, except for a few cases where general self-organization patterns were qualitatively computed^9^,^10^. These challenges have driven the development of morphology characterization techniques.

Standard BHJ characterization methods such as electron or X-ray microscopy/spectroscopy potentially provide an adequate spatial resolution and even the possibility to reconstruct the three-dimensional BHJ structure^11^,^12^ by contrast enhancement techniques^13^ such as energy filtering^14^ and special sample preparation, including selective staining of one of the materials^15^. Sub-10 nm spatial resolution, however, is not always easily achieved due to the typically low-contrast combinations of donor:acceptor materials used in organic photovoltaics. Another powerful method to characterize the morphology is atomic force microscopy (AFM) with typical 10 nm spatial resolution^16^. However, AFM provides information only about the surface topography, which is not necessarily representative for the bulk morphology^17^.

Aiming to overcome these limitations, complementary methods to control and optimize the morphology have been developed based on spectroscopic approaches, e.g. monitoring photoluminescence (PL) of interfacial charge-transfer states^18^, or measuring exciton diffusion by PL quenching^19^ or pump-probe spectroscopy^20^,^21^,^22^. These methods are mainly focused on diffusion of the excitons in the polymer domains, which has been shown to provide valuable information on the polymer and/or fullerene domain sizes^19^,^21^. The sensitivity of these methods to determine the domain sizes is essentially limited to the delocalization size of polymer excitons, i.e. several repeating polymer units, or 5–10 nm^23^,^24^.

Modern OSCs comprise high loadings of highly absorptive C_70_-based fullerene acceptors (up to 98%)^25^, which makes the fullerene absorption comparable or even higher than the absorption of the polymer. Consequently, a significant fraction of separated charges is generated after dissociation of the fullerene excitons via hole-transfer (HT) process^26^,^27^. Large fullerene domains readily observable with AFM^28^,^29^ exceed by far the fullerene exciton diffusion length (<10 nm)^30^,^31^ which results in significant losses in exciton harvesting. On the other hand, too fine polymer-fullerene intermixing (which is hard to observe by conventional characterization techniques) leads to increased non-geminate recombination of charges and lack of pathways to the electrodes for charge extraction^32^,^33^. Therefore, the fullerene phase morphology is as decisive for high efficiency OSCs as the polymer one, thereby calling for simple yet reliable characterization methods.

In the fullerene domains, the excitons are delocalized very moderately^34^,^35^. Therefore, the averaged time needed for the exciton to reach the donor-acceptor interface is determined by the fullerene domain size. The information about blend morphology can be extracted by excitation of the fullerene acceptor (e.g. soluble C_70_ derivative PC_71_BM), and detecting the time that is taken for the exciton to diffuse to the fullerene-polymer interface where it dissociates into separated charges^35^,^36^ (Fig. 1). The latter are detected by e.g. the charge-induced (polaron) photo-induced absorption (PIA)^37^ caused by the presence of positive charges (holes) at the polymer backbone. A number of previous studies^27^,^38^,^39^,^40^,^41^,^42^,^43^ have reported correlations between fullerene exciton dynamics and blend morphology; however, no attempt has been made to extract any information on the fullerene domain size.

In this paper, we use PIA for characterization of the nanoscale morphology of the fullerene domains in organic BHJs. We show for three polymers, regiorandom (RRa) P3HT, regioregular (RRe) P3HT and MDMO-PPV, selected as benchmark materials for exemplary cases of BHJ morphologies, that their blends with PC_71_BM contain the mixed phase with PC_71_BM domains typically up to 15 nm in size. The presence of large (>50 nm) PC_71_BM domains is also evidenced by a reduction of the efficiency of photon-to-charge harvesting. The blend composition was independently verified by GISAXS/GIWAXS, AFM, TEM/SEM and time-resolved photoluminescence (PL) techniques, with perfect matching of all the results. Unique spectroscopic signatures of subtle changes in BHJ morphology observed herein hold great promise for applications of the proposed technique for on-the-fly characterization of fully functional devices.

## Results

### Exciton dissociation dynamics

Spectrally-selective excitation of PC_71_BM followed by spectrally-selective probing of the polymer allows spatial decoupling of the excitation and the probing processes and therefore obtaining the exciton travelling time. Selective photoexcitation of PC_71_BM was achieved by tuning the excitation wavelength below the bandgap of the polymer where PC_71_BM has a significantly higher absorption coefficient (680 nm for RRa-P3HT and RRe-P3HT, and 630 nm for MDMO-PPV, see Supplementary Section 1 for details).

PC_71_BM exciton dissociation into charges was monitored by probing the charge-induced (polaron) PIA of the polymers in the mid-IR region^44^,^45^. For this, the wavelength of the probe IR pulse was set close to the maximum of the low-energy polaron band at ~3 μm for all three polymers (see Supplementary Section 2 for details). As the exciton is harvested (i.e. reaches the interface), the polaron absorption increases proportionally to the amount of charges (holes) at the polymer. By changing the delay between the excitation and the probe pulses, exciton diffusion preceding exciton dissociation is monitored in the real time^35^,^36^. Note that after having reached the interface, the excitons do not necessary produce free charges but also the charge-transfer (CT) states which eventually either dissociate into free charges or geminately recombine in a ns timescale^39^,^46^,^47^,^48^,^49^. However, from the point of view of the PIA response, the exact route of the exciton dissociation makes no difference as even the (interfacially) bound charges produce a similar PIA signal^50^. Therefore, in both cases the gradual build-up of the PIA signal reflects the diffusion time needed for the PC_71_BM excitons to reach the interface.

Dynamics of the exciton dissociation into charges are shown in Fig. 2 for the blends with different PC_71_BM weight ratios. The transients at low and high PC_71_BM loads were corrected for the weak pristine polymer response due to the finite excitation contrast, and IR response of the PC_71_BM excitons, respectively (see Supplementary Section 3). All transients were also normalized to the PC_71_BM absorption at the excitation wavelength so that the transient amplitudes represent the charge yield per absorbed photon (i.e. exciton harvesting efficiency) to allow for direct comparison of the transient amplitudes at different PC_71_BM loads.

The exciton harvesting dynamics for the blends with the three polymers have a number of similar features that can be summarized as follows: for the blends with low PC_71_BM content, the transients exhibit a large amplitude and a rapid rise time (<1 ps), whereas for the blends with high PC_71_BM content the amplitudes are decreased (except of the RRa-P3HT blends) while the rise of the response becomes substantially slower, up to 100 ps. The latter dynamics are assigned to the PC_71_BM exciton diffusion followed by the dissociation to charges at the PC_71_BM-polymer interface via hole-transfer process^26^,^40^,^42^. We attribute increasing rise time to variations in the PC_71_BM domain size: the larger the PC_71_BM domains, the longer it takes for excitons to reach an interface and more excitons are lost.

The exciton harvesting dynamics are quite analogous in MDMO-PPV- and RRa-P3HT-based blends with low PC_71_BM content (<40%). The similar timescale of the initial signal build-up combined with the close-to-unity amplitudes point to the nanomorphology of the mixed phase with a phase separation of ~10 nm. At higher PC_71_BM concentrations a dramatic drop of the signal amplitude is observed with the signal reducing to naught at >70% PC_71_BM concentration. This indicates the formation of large PC_71_BM domains^51^ with the size much larger that the exciton diffusion length (i.e. ~10 nm) separated from the mixed phase. The sharp decrease of the signal amplitude points to an increase of the volume fraction of the large domains, which reaches almost 100% in blends with >70% PC_71_BM content (i.e. the polymer and fullerene phases are fully separated).This is consistent with the known property of the MDMO-PPV-based blends to form large fullerene domains above a certain acceptor weight fraction^52^,^53^,^54^.

For the RRe-P3HT-based blends, the exciton dissociation dynamics are different. For low PC_71_BM content (<40%), the initial build-up of the signal is significantly faster as compared to RRa-P3HT- and MDMO-PPV-based blends. This indicates extremely fine intermixing of polymer and PC_71_BM in the mixed phase, probably even isolated PC_71_BM molecules dispersed in the polymer matrix. At higher PC_71_BM loadings, the initial build-up slows down indicating the coarser intermixing within the mixed phase. Simultaneously, the decrease of the exciton harvesting is observed, similarly to the MDMO-PPV blends, but to a significantly smaller extent. The observed difference in dynamics between the RRa-P3HT and the RRe-P3HT originates from the different morphology: the blends with RRa-P3HT are completely amorphous while in blends with RRe-P3HT semi-crystalline domains of RRe-P3HT are formed prior to the aggregation of PC_71_BM^55^. Hence, the PC_71_BM molecules are pushed outside the RRe-P3HT nanocrystals^56^ to aggregate into the domains. Therefore, we assign exciton losses (Fig. 2c) in the blends of RRe-P3HT with 40–60% of PC_71_BM to the formation of the PC_71_BM domains with sizes much larger than the exciton diffusion length.

At 70% of PC_71_BM, the exciton harvesting suddenly increases which indicates an abrupt change in the RRe-P3HT:PC_71_BM nanostructure. Simultaneously, around these blend compositions the absorption shoulder in the red spectral region, which is associated with the absorption by the RRe-P3HT nanocrystals, vanishes (see Supplementary Section 4). Additionally, the GIWAXS data show a significant change of the blend morphology at 70% PC_71_BM contents (see Supplementary Section 5). All these point to disruption of the RRe-P3HT nanocrystals^57^,^58^ for high PC_71_BM load^57^.

The results of PIA measurements were independently verified by the time-resolved PL quenching technique^19^,^30^ (Supplementary Section 7). Due to intrinsic limitations of the PL technique such as spectral overlap of PL from PC_71_BM, polymers and CT states, and limited time resolution (~5 ps), it is nearly impossible to quantitatively characterize the PC_71_BM domain sizes. Nonetheless, the case of MDMO-PPV-based blends allows the direct comparison of PL quenching efficiency with exciton PIA harvesting efficiency, to produce an excellent match (Supplementary Section 7, Supplementary Figure 13). This lends additional support to the proposed PIA method.

Summarizing the discussion above: despite some similarities, the three PC_71_BM:polymer blends exhibit very different exciton harvesting dynamics as a function of the blend composition. Interestingly, the exciton harvesting is sensitive to subtle changes in the morphology as is for instance shown by the changes of the dynamics upon disappearance of nanocrystals in RRe-P3HT. This clearly indicates that a more detailed insight in the characteristic sizes of the mixed phases and the PC_71_BM domains can be obtained through modeling of the experimental data.

### Characterization of the nanomorphology from Monte Carlo simulations

Monte Carlo (MC) simulations for modeling exciton dynamics have the important advantage over analytical description^4^,^5^,^30^,^59^ that they allow for inclusion of energetic disorder^60^,^61^, which cannot be neglected for the solvent-processed materials and blends. We modeled the exciton diffusion as random hopping in disordered PC_71_BM domains of a mixed phase of spherical domains with diameter *d*_*m*_ and large domains with larger diameter *d*_*c*_ and a volume fraction *f* (see the Methods section for details).

The MC simulations reproduce the experimental data fairly well (Fig. 2, solid lines). The estimated domain size in the mixed phase varies from 2 nm to 15 nm depending on the blend composition and the particular polymer (Fig. 3). In the low (<40%) PC_71_BM load blends only the mixed phase is present with typical PC_71_BM domain sizes of 6–8 nm in amorphous RRa-P3HT and MDMO-PPV polymers, and 2–3 nm in RRe-P3HT-based blends. With the increase of PC_71_BM load, coarsening of the mixed phase is observed in RRa-P3HT and RRe-P3HT based blends. In contrast, in MDMO-PPV blends higher PC_71_BM load does not result in any significant change in domain sizes of the mixed phase but leads to explosive growth of extremely large PC_71_BM domains (up to 1 μm, Fig. 4), which results in dramatic decrease of the PIA signal (Fig. 2b). Interestingly, the typical domain size of the mixed phase does not significantly increase with increasing of PC_71_BM content even when the large domains begin to form. In contrast, in MDMO-PPV blends with high PC_71_BM content the fine mixed phase coexists with the separated PC_71_BM domains, which volume share depends on PC_71_BM concentration (Fig. 4a).

The sizes of large PC_71_BM domains obtained from the MC simulations and independently from AFM for the MDMO-PPV blends and from TEM/GISAXS for the RRe-P3HT blends (see Supplementary Sections 5, 6 and 8–10) are summarized in Fig. 4b. MC simulations yield only the minimal sizes of the large PC_71_BM domains from the long-time exciton harvesting dynamics, while the volume fraction is straightforwardly obtained from decrease of the signal amplitude. The sizes of the large domains derived from the MC simulations match reasonably well the independently measured values for the RRe-P3HT based blends. In the MDMO-PPV case the deviations are quite substantial because for the proposed technique 0.1 and 1 μm size domains look identical as no excitons are harvested from either of them. Note, however, that the PC_71_BM domain sizes in the mixed phase (Fig. 3) lay below either attainable resolution (AFM) or contrast (TEM) of the conventional methods but are readily captured by spectroscopic means.

For the P3HT:PC_71_BM blends with high PC_71_BM loadings (>40%), the blend composition was also independently verified by GISAXS and GIWAXS (Supplementary Sections 5, 6)^62^. For the mixed phase, the domain sizes of 2 nm and 15 nm with different shares were observed which is in good agreement with the current two-domain model (Fig. 3c). The sizes of large domains were estimated from GISAXS as >100 nm which matches perfectly both results obtained by TEM and from MC simulations (Fig. 4b).

### Influence of energetic disorder

With the MC machinery in hand, we studied the influence of energetic disorder on the exciton losses in large PC_71_BM domains, by performing simulations of exciton harvesting from 1–100 nm domains (Fig. 5a, red line) with and without disorder. If the phase intermixing is fine (<15 nm, i.e. similar to the mixed phase), almost 100% of excitons are harvested in both cases, i.e. the disorder does not play any crucial role. The reason is two-fold. First, with such small domain sizes, the significant fraction of excitons is generated in the close proximity to the interface and dissociate into charges immediately with 100% efficiency. Second, even those excitons that are generated deeper in the PC_71_BM domains, reach the interface faster (in ~400 ps for 15 nm domain size, Fig. 5b) than the exciton lifetime of 650 ps.

In contrast, the exciton harvesting efficiency rapidly decreases in the large domains (>50 nm), because the exciton diffusion time needed to reach the interface becomes comparable with the exciton lifetime. This is a direct consequence of ten-fold decrease of the exciton diffusion coefficient within 100 ps (Fig. 5b). The diffusion coefficient at long times (>100 ps) changes only insignificantly and amounts to *D* ~ 1.5∙10^−4^ cm^2^/s, which is consistent with the earlier report^30^. This value can be safely used to estimate the exciton diffusion length since the exciton lifetime (650 ps) is much larger than the time needed for diffusion coefficient equilibration (~100 ps). However, the exciton dynamics at the early times (<10 ps) are determined by the highly non-equilibrium *D* (Fig. 5b) which explains fast exciton harvesting from the mixed phase (<10 ps for 3 nm domain size).

In materials with negligible energy disorder, where the diffusion coefficient does not depend on time, the excitons are harvested extremely efficiently (>75%) even from the PC_71_BM domains as large as 100 nm (Fig. 5a, blue line). This fact explains high efficiency of vacuum-deposited TPTPA/C_70_ solar cells with >95% content of C_70_ with extremely low disorder of 5 meV^25^,^35^. Interestingly, in the disordered medium exciton harvesting is limited not only by dynamical decrease of *D*, but also by the presence of low-energy trap sites. As the result, the exciton is trapped at a low-energy site for a long time, which significantly decreases the diffusion length (Supplementary Section 11). Thus, even though the excitons can be effectively harvested in a BHJ based on disordered materials with fine phase intermixing, decreasing of the energy disorder seems to be more favorable for the blend optimization as in this case larger domain sizes lead to smaller interface area and, therefore, decreased non-geminate charge recombination.

## Discussion

The exciton harvesting dynamics from the PC_71_BM phase have been successfully obtained by a PIA technique and modeled by the Monte-Carlo simulations to yield valuable information on the BHJ morphology. The BHJ blends studied herein contain mixed-phase PC_71_BM domains of the size of several nanometers (up to 15 nm) as well as the large PC_71_BM domains with sizes exceeding 50 nm. These findings are fully consistent with the paradigm of a hierarchical BHJ morphology^56^,^63^,^64^ and were independently confirmed by GISAXS/GIWAXS, AFM, TEM/SEM and time-resolved PL measurements. Note that due to a number of fundamental limitations, the PL technique is not capable to deliver similar quantitative information.

Significant differences of BHJ morphology in terms of formation of the mixed phase and the large (>50 nm) PC_71_BM domains have been observed for the blends with donor polymers of RRa-P3HT, MDMO-PPV, and RRe-P3HT. The phase separation of the mixed phase varies from 2 to 15 nm in PC_71_BM:RRa/RRe-P3HT blends and is ~7 nm in PC_71_BM:MDMO-PPV blends. RRa-P3HT based blends demonstrate fine intermixing without large PC_71_BM domains within the whole range of PC_71_BM loads investigated. In contrast, the MDMO-PPV and RRe-P3HT based blends exhibit the formation of large PC_71_BM domains. Observed disruption of the RRe-P3HT nanocrystals at the PC_71_BM load from 60% to 70%, verified by GIWAXS, underlines the high sensitivity of the technique used. We have also demonstrated that the exciton losses in the large PC_71_BM domains are related to a high energetic disorder of ~70 meV and in particular to the low-energy trap sites. Decreasing the energetic disorder (e.g. by applying vacuum deposition techniques)^35^ dramatically improves the harvesting efficiency from the fullerene domains. This suggests that increasing the material order is a winning strategy in the exciton harvesting optimization.

The main simplification in the MC simulations is an assumption of two types of the spherically shaped domains. Although realistic morphology is much more complex^30^,^52^, this simple model captures the essential aspects of the PC_71_BM morphology, with two different kinds of domains being among them. Modern computational methods of predicting more realistic BHJ patterns^9^,^10^ could readily incorporate the MC approach used herein. Next, the domain size of the mixed phase can be slightly underestimated due to the exciton delocalization among 4–5 PC_71_BM molecules^34^,^35^. Additionally, the possibility of the long-range hole transfer from next to the outer layer of PC_71_BM domains^65^,^66^ cannot be ruled out. The observed increase of hole transfer time with increasing of PC_71_BM content and therefore domain size (Supplementary Section 12) is in line with this supposition. Nevertheless, all latter effects do not have serious influence on the results and could be readily accounted for in the MC simulations.

Another concern is the possible dependence of the kinetic parameters (i.e. exciton lifetime, hopping time and the disorder) on the PC_71_BM domain size. We thoroughly tested stability of domain sizes retrieval for both mixed phase and large domains with respect to the kinetic parameters of the model (Supplementary Section 13) and found that vast variations of them do not result in substantial changes of the PC_71_BM domain sizes. This is attributed to extremely fast extraction of the excitons from the mixed phase. Therefore, the obtained domain sizes for the mixed phase are reliable even if the kinetic parameters are different from the bulk PC_71_BM. On the other hand, the large PC_71_BM domains behave as the bulk material so that the parameters derived from the PL measurements can be safely used.

The charge generation after excitation of PC_71_BM is especially important for modern solar cells involving narrow bandgap polymers, where high PC_71_BM loadings are used and the fullerene becomes the main absorber in the green-blue region of the spectrum. In this work, we used the polymers with relatively wide bandgap to selectively excite PC_71_BM and therefore to simplify the analysis to demonstrate the proof of concept. In the case of modern low-bandgap donors, selective excitation of PC_71_BM is hardly achievable even in the blue where PC_71_BM absorption increases, and both donor and PC_71_BM PIA responses have to be considered. As the excitons from donor phase dissociate at a 100-fs timescale^40^,^42^,^46^,^50^,^67^,^68^, i.e. significantly faster than any diffusion-delayed exciton dissociation, the donor PIA response can be considered as step-like function (for an example of such retrieval, see Supplementary Section 14). In addition, transient anisotropy might be used as an extra contrast parameter to distinguish between the donor and acceptor PIA responses^26^,^47^. Overall, the proposed method constitutes a first step towards PIA spectroscopy as a tool that provides a valuable feedback on optimization of BHJ morphology and can be expanded to modern donor materials such as more efficient polymers^1^,^42^,^69^ and small organic molecules^39^,^70^,^71^.

## Methods

### Materials and sample preparation

Poly[2-methoxy-5-(3′,7′-dimethyloctyloxy)-1,4-phenylenevinylene] (MDMO-PPV), regiorandom and regioregular poly(3-hexylthiophene-2,5-diyl) (P3HT) were purchased from Sigma-Aldrich. Regioregular P3HT had regioregularity greater than 90% head-to-tail regiospecific conformation. The soluble C_70_ derivative [6,6]-Phenyl C_71_ butyric acid methyl ester (a mixture of isomers)^72^ (PC_71_BM), purity >99%by HPLC with respect to the total fullerene content was purchased from Solenne.

Blends of MDMO-PPV and both P3HTs were prepared with PC_71_BM weight content ranging from 10% to 90%. The preparation procedure was the following: the polymer and PC_71_BM were dissolved separately with concentrations of 3 g/L for MDMO-PPV and 10 g/L for RRa/RRe-P3HT in 1,2-Dichlorobenzene (ODCB) and stirred overnight on the hot plate with temperature of 60°. The solution of PC_71_BM was filtered using polytetrafluoroethylene (PTFE) filter with pore size of 0.2 μm. The solutions of polymer and fullerene were mixed with the appropriate volumes to obtain 10–90% PC_71_BM content in the solution. The final solutions were drop cast by equal volumes of 0.2 ml on the glass microscope cover slides with the thickness of 150 μm, and were allowed to dry for several hours making the solvent-assisted annealing^73^,^74^. During all measurements, the samples were kept under the nitrogen atmosphere to prevent their degradation; none was observed. Linear absorption was measured using standard Perkin Elmer Lambda 900 spectrometer. Film thicknesses were measured using Dektak profilometer.

### PIA measurements

Time-resolved photoinducedabsorption (PIA) spectroscopy was performed in a home-built Ti:Sapphire-based setup. The output of a 1 kHz amplifier was split to pump a noncollinear optical parametric amplifier (NOPA)^75^ and a 3-stages IR OPA^76^. NOPA was producing visible 30 fs, 40 μJ pulses with the wavelength tunable in the range of 500–700 nm. The excitation wavelength was selected for the best absorption ratio between fullerene and polymer: for PC_71_BM:RRa/RRe-P3HT and PC_71_BM:MDMO-PPV as 680 nm and 630 nm, respectively. For the MDMO-PPV blends, the wavelength was set slightly off the maximal contrast point (630 nm *vs.* 650–660 nm, Supplementary Figure 1) in the region of higher PC_71_BM absorption to increase the signal-to-noise ratio. The IR OPA generated ~80 fs probe pulses at 3.3 μm wavelength suitable for probing the low-energy polaron absorption (see Supplementary Section 2). The visible pump was focused into a factor of 2 wider spot than the IR probe to minimize the spatial inhomogeneity of the pump.

The PIA response was calculated as the relative transmission change *ΔT/T*, where *T* and Δ*T* stand for transmission and transmission change with and without the excitation pulse, respectively. Pump flux was carefully attenuated with gradient neutral density filter for the PIA response to be in the linear regime (75 μJ/cm^2^ for the P3HT blends and 120 μJ/cm^2^ for the MDMO-PPV blends). Note that due to the extremely low absorption of the samples at the excitation wavelengths, the absorbed photon density was below 10^−3^ photons/nm^3^ (i.e. ~1 photon per 10 nm of length) which minimizes bi-exciton annihilation to nihil (see Supplementary Section 15).

The polarization of the probe beam was rotated by 45° by the half-wave plate with respect to the pump. The beamsplitter was placed after the sample to detect parallel and perpendicular components of IR probe polarization with respect to the pump polarization with two wire-grid polarizers (1:100 extinction) and indium antimonide (InSb) photodiodes. PIA signals with parallel and perpendicular polarizations were used to recalculate isotropic component using the following relation^77^:


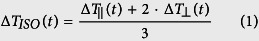


where the indices || and ⊥ denote parallel and perpendicular components, and *t* is a time delay. The third InSb detector was used as a reference for IR pulses to enhance the signal-to-noise ratio of the PIA signal.

The precise pump-probe time-overlap position (zero delay) was carefully checked before and after each scan (every 30 minutes) by measuring the reference sample, a blend of poly[2-methoxy-5-(2-ethylhexyloxy)-1,4-phenylenevinylene] (MEH-PPV) mixed with 2,4,7-trinitrofluorenone (TNF) by weight ratio of 1:0.3. This blend forms a ground-state charge transfer complex with the apparatus-limited PIA response^78^,^79^,^80^. The materials were dissolved in chlorobenzene 2 g/L separately and mixed together. The final solution was drop-cast from chlorobenzene solution of2 g/L on the same substrate as samples and allowed to dry. The root-mean-square (rms) drift of the reference zero delay was ~5 fs during 15-hour measuring sessions.

### Monte-Carlo simulations

**Figure 1:**
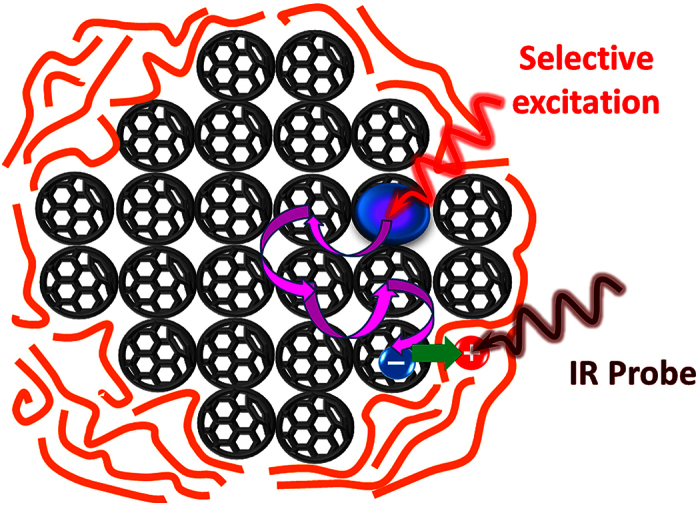
Conceptual representation of the spectroscopic technique used. The PC_71_BM phase is selectively excited by the ultrafast laser pulse (red). The photogenerated exciton (the blue-purple circle) diffuses to the interface where it dissociates into charges via hole transfer (green straight arrow) to the polymer phase (orange spaghetti). The number of accumulated holes at the polymer phase is probed by a delayed IR probe pulse via polaron-induced absorption.

**Figure 2:**
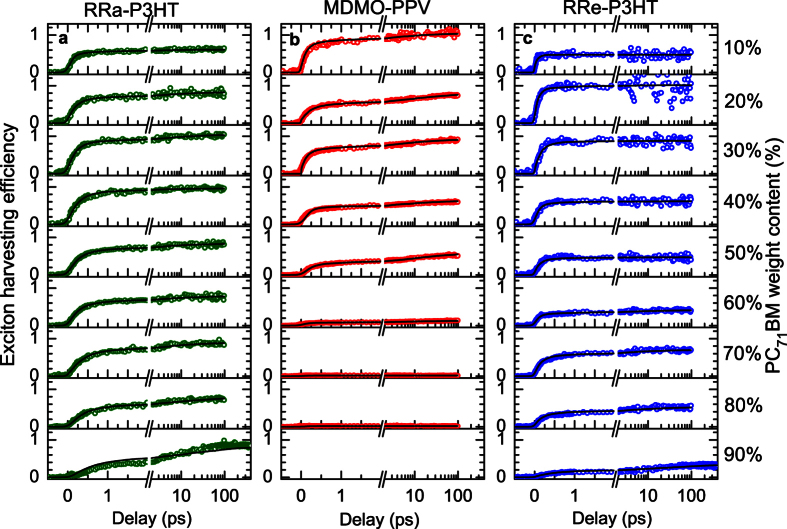
Exciton harvesting dynamics. Normalized exciton harvesting efficiency as a function of excitation-probe delay for blends with different PC_71_BM weight fractions with (**a**) RRa-P3HT, (**b**) MDMO-PPV, and (**c**) RRe-P3HT. Symbols represent experimental data points after the polymer and PC_71_BM background subtraction and normalization to the PC_71_BM absorption at the excitation wavelength; the lines show the results of Monte-Carlo simulations. For each series, the charge yield was scaled to the maximal amplitude where unity relative efficiency of exciton harvesting was assumed (see Supplementary Section 3 for details). The 90% MDMO-PPV sample produced no polaron response and therefore the respective transient is not shown.

**Figure 3:**
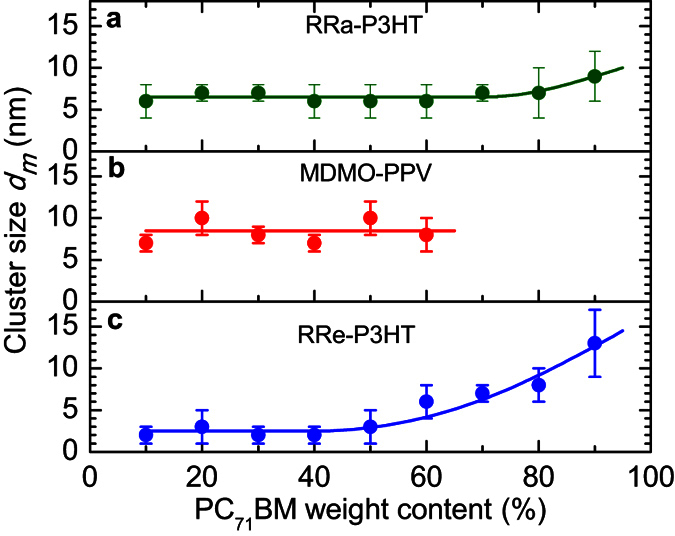
Domain sizes for different blends. Domain size *d*_*m*_ of the mixed PC_71_BM:polymer phase as a function of the blend composition for RRa-P3HT (**a**), MDMO-PPV (**b**) and RRe-P3HT (**c**) blends. Symbols are results of Monte-Carlo simulations while the solid lines are guides to the eyes. Error bars are derived from uncertainty of the Monte-Carlo simulations.

**Figure 4:**
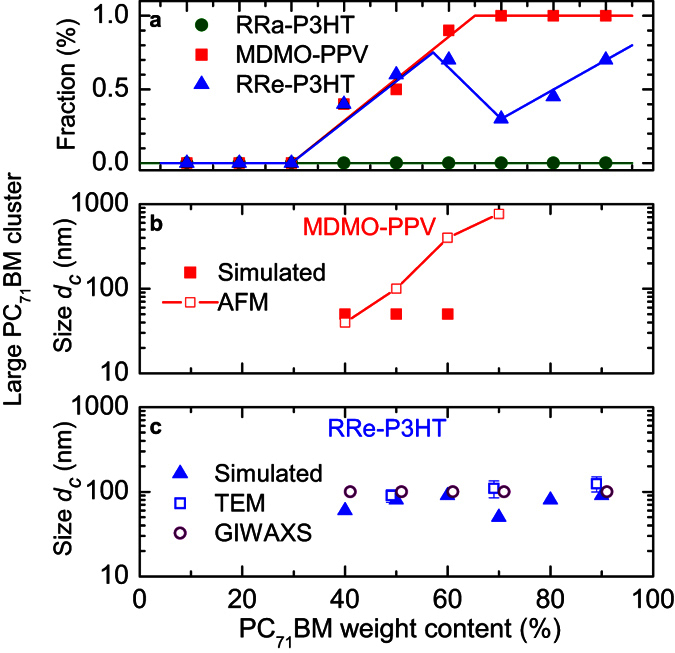
Fraction and sizes of large domains in different blends. Volume fraction of the PC_71_BM domains (**a**) and sizes of the PC_71_BM large domains for MDMO-PPV (**b**) and RRe-P3HT (**c**) as obtained from the MC simulations (solid symbols). The solid symbols represent the minimal values obtained from MC simulations while the open symbols represent experimental data obtained from the AFM (red squares), TEM (blue triangles) and GIWAXS (purple circles) measurements (see Supplementary Sections 5–10). The GIWAXS data represent the minimal size of the PC_71_BM domains. Solid lines are guides to the eye. The data in (**c**) were slightly displaced from the exact concentration for clarity.

**Figure 5:**
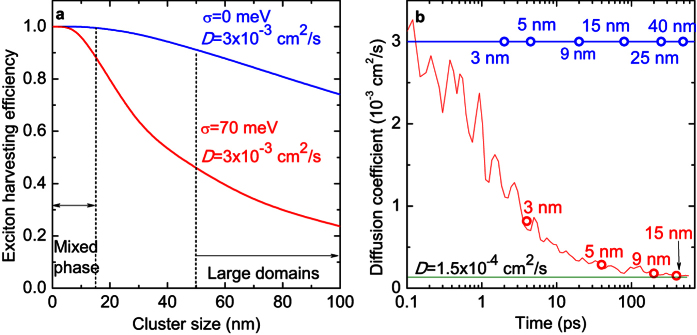
Influence of energetic disorder on exciton diffusion process. (**a**) Simulated exciton harvesting efficiency *vs.* domain size at room temperature. The harvesting efficiencies for *D*_*0*_ = 3 ∙ 10^−3^ cm^2^/s (which corresponds to the initial diffusion coefficient in PC_71_BM obtained from simulations) with and without energetic disorder are shown in red in blue, respectively. The areas of the mixed phase and large domains are also indicated. (**b**) Simulated exciton diffusion coefficient dependence on time for energy disorder of *σ* = 70 meV (red line) and without energy disorder (blue line). In the case of disorder, the diffusion coefficient decreases due to the exciton energy downhill migration as subsequent trapping. Stabilized diffusion coefficient of *D* = 1.5 ∙ 10^−4^ cm^2^/s is shown by green line. Open circles indicate the domain size from which the excitons are extracted at the given time.

In the MC simulations, the mixed phase was modelled as spherical PC_71_BM domains surrounded by the polymer (Fig. 1). The coexistence of the mixed phase and large PC_71_BM domains^81^ was taken into account by including two types of domains with different sizes. Exciton diffusion was simulated as random hopping between discreet PC_71_BM molecules in the cubic grid cells. The boundaries of the domains were determined as spheres of variable diameters *d*_*m*_(for the mixed phase)and *d*_*c*_(for the large PC_71_BM domains), *d*_*m*_<*d*_*c*_.

Energetic disorder of the potential energy landscape of PC_71_BM was taken into account by a Gaussian disorder model^82^. Energies within the Gaussian distribution, with standard deviation *σ,* were randomly assigned to the PC_71_BM molecules. Initially, an exciton with finite effective lifetime *T*_*1*_ is randomly placed in one of the two domains with probability *f* which reflects the volume ratio of the mixed phase to the large domains. At each step, the exciton hops into a random direction by one grid point with hopping time *τ*and hopping probability *p*_*ij*_ which depends on the energies of the starting *E*_*i*_ and target *E*_*j*_ grid points:


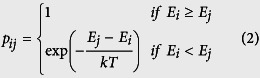


where *kT* is the Boltzmann factor. Finally, exciton dissociation into charges at the surface of PC_71_BM domain occurs with a finite HT time *τ*_*ht*_^26^, after which the resulting hole begins to contribute to the PIA signal. The weak contribution of PC_71_BM excitons was taken into account by assigning it the relative cross-section *α* with respect to the hole response (see Supplementary Section 3). The total PIA signal was convoluted with a Gaussian apparatus function of 70–100 fs width.

The exciton lifetime *T*_*1*_, hopping time *τ* and energy disorder parameter *σ* were obtained independently from the photoluminescence data of PC_71_BM films with TPTPA quenchers as *T*_*1*_ = 650 ps, *τ* = 0.3 ps, and *σ* = 70 meV (see Supplementary Sections 16, 17). The fact that a single set of kinetic parameters is needed to describe as broad range of quencher concentrations as 0.0125–50%, signifies similar exciton diffusion in PC_71_BM domains of different sizes. Therefore, the remaining fit parameters for each sample are the PC_71_BM domain sizes *d*_*m*_ and *d*_*c*_, the hole transfer time *τ*_*ht*_ and the volume fraction of the large domains *f*. Each of the four fit parameters is responsible for the particular feature of the PIA transients which makes them independent in the fitting procedure. The early-time dynamics (<0.5 ps) are mainly driven by the HT process characterized by the hole transfer time *τ*_*ht*_ (see Supplementary Section 11). The intermediate time window (1–10 ps) accounts for exciton dissociation from the mixed phase and is determined by the size of small domains *d*_*m*_. Size of the large domains *d*_*c*_ is responsible for the later dynamics (>10 ps), while the volume fraction *f* determines the PIA transient amplitude. To collect the necessary statistics, each simulation was run 1000 times for each sample (3000 times for MDMO-PPV:PC_71_BM blend with 60% PC_71_BM content). The dependence of the three-dimensional exciton diffusion coefficient on time was obtained from the exciton displacement as:


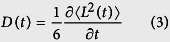


where L(t) is the exciton displacement and <> denotes averaging over the whole exciton ensemble.

### TEM measurements

For the TEM measurements, free-standing thin films were prepared in a clean room using the procedures described in refs [83 and 84] (see Supplementary Section 10 for details). The 400 mesh copper grids were used to pick up the freestanding films from water and put into Philips CM120 electron microscope operating at 120 keV. Just before performing the TEM measurement, all PC_71_BM:RRe-P3HT films were stained with iodine vapors for several minutes to improve the contrast between PC_71_BM and P3HT^15^.

### GISAXS and GIWAXS measurements

GISAXS and GIWAXS measurements were performed on the custom made MINA X-ray instrument built on a Cu rotation anode (λ = 1.5413 Å). GISAXS measurements were performed using a sample-to-detector distance of 3 m. 2D patterns have been collected using a Vantec2000 detector (2048 × 2048 pixels array with pixel size 68 × 68 microns). Samples were aligned using Huber motors at an incident angle of *α*_*i*_ = 0.3°. The in-plane GISAXS intensity cuts (along the 2*θ*_*f*_ or the *q*_*y*_ direction) were calculated at the Yoneda peak height (α_f_ = 0.18°). The direct beam center position on the detector and the sample-to-detector distance were calibrated using the diffraction rings from a standard Silver Behenate powder. As the incident angle was kept fixed during the measurements, transmission functions reduce to constant values and the in-plane intensity measured directly the scattering factor of the objects inside the film.

GIWAXS measurements were performed using a sample-to-detector distance of 130 cm. 2D patterns have been collected using a Vantec500 detector (1024 × 1024 pixels array with pixel size 136 × 136 microns). The direct beam center position on the detector and the sample-to-detector distance were calibrated using the diffraction rings from a standard Silver Behenate powder. Integrated intensities have been obtained by radially averaging of the intensity with respect to the beam center using the GIXGUI software^85^.

## Additional Information

**How to cite this article**: Serbenta, A. *et al*. Bulk heterojunction morphology of polymer:fullerene blends revealed by ultrafast spectroscopy. *Sci. Rep.* **6**, 36236; doi: 10.1038/srep36236 (2016).

**Publisher’s note:** Springer Nature remains neutral with regard to jurisdictional claims in published maps and institutional affiliations.

## Supplementary Material

Supplementary Information

## References

[b1] He Z. . Single-junction polymer solar cells with high efficiency and photovoltage. Nature Photon. 9, 174–179 (2015).

[b2] You J. . A polymer tandem solar cell with 10.6% power conversion efficiency. Nat. Commun. 4, 1446 (2013). 23385590 10.1038/ncomms2411 PMC3660643

[b3] Su Y.-W., Lan S.-C. & Wei K.-H. Organic photovoltaics. Mater. Today 15, 554–562 (2012).

[b4] Menke S. M. & Holmes R. J. Exciton diffusion in organic photovoltaic cells. Energy Environ. Sci. 7, 499–512 (2014).

[b5] Mikhnenko O. V., Blom P. W. M. & Nguyen T.-Q. Exciton diffusion in organic semiconductors. Energy Environ. Sci. 8, 1867–1888 (2015).

[b6] Hiramoto M., Fujiwara H. & Yokoyama M. Three-layered organic solar cell with a photoactive interlayer of codeposited pigments. Appl. Phys. Lett. 58, 1062–1064 (1991).

[b7] Halls J. J. M. . Efficient photodiodes from interpenetrating polymer networks. Nature 376, 498–500 (1995).

[b8] Yu G., Gao J., Hummelen J. C., Wudl F. & Heeger A. J. Polymer photovoltaic cells: Enhanced efficiencies via a network of internal donor-acceptor heterojunctions. Science 270, 1789–1791 (1995).

[b9] Lee C. K., Pao C. W. & Chu C. W. Multiscale molecular simulations of the nanoscale morphologies of P3HT:PCBM blends for bulk heterojunction organic photovoltaic cells. Energ. Environ. Sci. 4, 4124–4132 (2011).

[b10] Kouijzer S. . Predicting morphologies of solution processed polymer:fullerene blends. J. Am. Chem. Soc. 135, 12057–12067 (2013). 23863101 10.1021/ja405493j

[b11] Andersson B. V., Herland A., Masich S. & Inganäs O. Imaging of the 3D nanostructure of a polymer solar cell by electron tomography. Nano Lett. 9, 853–855 (2009). 19119912 10.1021/nl803676e

[b12] Herzing A. A., Richter L. J. & Anderson I. M. 3D nanoscale characterization of thin-film organic photovoltaic device structures via spectroscopic contrast in the tem 1. J. Phys. Chem. C 114, 17501–17508 (2010).

[b13] Chen W., Nikiforov M. P. & Darling S. B. Morphology characterization in organic and hybrid solar cells. Energy Environ. Sci. 5, 8045–8074 (2012).

[b14] Masters R. C. . Sub-nanometre resolution imaging of polymer-fullerene photovoltaic blends using energy-filtered scanning electron microscopy. Nat. Commun. 6, 6928 (2015). 25906738 10.1038/ncomms7928 PMC4423221

[b15] Dimitrov S. D. . Efficient charge photogeneration by the dissociation of PC70BM excitons in polymer/fullerene solar cells. J. Phys. Chem. Lett. 3, 140–144 (2012).

[b16] Fischer F. S. U. . Highly crystalline films of PCPDTBT with branched side chains by solvent vapor crystallization: Influence on opto-electronic properties. Adv. Mater. 27, 1223–1228 (2014). 25482608 10.1002/adma.201403475

[b17] Dang X.-D. . Nanostructure and optoelectronic characterization of small molecule bulk heterojunction solar cells by photoconductive atomic force microscopy. Adv. Funct. Mater. 20, 3314–3321 (2010).

[b18] Baran D. . Qualitative analysis of bulk-heterojunction solar cells without device fabrication: An elegant and contactless method. J. Am. Chem. Soc. 136, 10949–10955 (2014). 25003533 10.1021/ja503134j

[b19] Ruseckas A., Shaw P. E. & Samuel I. D. W. Probing the nanoscale phase separation in binary photovoltaic blends of poly(3-hexylthiophene) and methanofullerene by energy transfer. Dalton Trans. 45, 10040–10043 (2009). 10.1039/b912198f 19904431

[b20] Grancini G. . Transient absorption imaging of P3HT:PCBM photovoltaic blend: Evidence for interfacial charge transfer state. J. Phys. Chem. Lett. 2, 1099–1105 (2011).

[b21] Westenhoff S., Howard I. A. & Friend R. H. Probing the morphology and energy landscape of blends of conjugated polymers with sub-10 nm resolution. Phys. Rev. Lett. 101, 016102 (2008). 18764126 10.1103/PhysRevLett.101.016102

[b22] Muller J. G. . Ultrafast dynamics of charge carrier photogeneration and geminate recombination in conjugated polymer:fullerene solar cells. Phys. Rev. B 72 (2005).

[b23] Kaake L. G., Moses D. & Heeger A. J. Coherence and uncertainty in nanostructured organic photovoltaics. J. Phys. Chem. Lett. 4, 2264–2268 (2013). 10.1021/jp408700m 24070027

[b24] Mukamel S. Comment on “coherence and uncertainty in nanostructured organic photovoltaics”. J. Phys. Chem. A. 117, 10563–10564 (2013). 24032455 10.1021/jp4071086

[b25] Cheyns D., Kim M., Verreet B. & Rand B. P. Accurate spectral response measurements of a complementary absorbing organic tandem cell with fill factor exceeding the subcells. Appl. Phys. Lett. 104, 093302 (2014).

[b26] Bakulin A. A., Hummelen J. C., Pshenichnikov M. S. & van Loosdrecht P. H. M. Ultrafast hole-transfer dynamics in polymer/PCBM bulk heterojunctions. Adv. Funct. Mater. 20, 1653–1660 (2010).

[b27] Dimitrov S. D. . Towards optimisation of photocurrent from fullerene excitons in organic solar cells. Energy Environ. Sci. 7, 1037–1043 (2014).

[b28] Kouijzer S. . Predicting morphologies of solution processed polymer:fullerene blends. J. Am. Chem. Soc. 135, 12057–12067 (2013). 23863101 10.1021/ja405493j

[b29] Brabec C. J. . Polymer–fullerene bulk-heterojunction solar cells. Adv. Mater. 22, 3839–3856 (2010). 20717982 10.1002/adma.200903697

[b30] Hedley G. J. . Determining the optimum morphology in high-performance polymer-fullerene organic photovoltaic cells. Nat. Commun. 4, 2867 (2013). 24343223 10.1038/ncomms3867 PMC3905772

[b31] Cook S., Furube A., Katoh R. & Han L. Estimate of singlet diffusion lengths in PCBM films by time-resolved emission studies. Chem. Phys. Lett. 478, 33–36 (2009).

[b32] Baumann A. . Influence of phase segregation on recombination dynamics in organic bulk-heterojunction solar cells. Adv. Funct. Mater. 21, 1687–1692 (2011).

[b33] Scharber M. C. & Sariciftci N. S. Efficiency of bulk-heterojunction organic solar cells. Prog. Polym. Sci. 38, 1929–1940 (2013). 24302787 10.1016/j.progpolymsci.2013.05.001 PMC3837184

[b34] Bernardo B. . Delocalization and dielectric screening of charge transfer states in organic photovoltaic cells. Nat. Commun. 5, 3245 (2014). 24488203 10.1038/ncomms4245

[b35] Kozlov O. V. . Real-time tracking of singlet exciton diffusion in organic semiconductors. Phys. Rev. Lett. 116, 057402 (2016). 26894732 10.1103/PhysRevLett.116.057402

[b36] Dowgiallo A.-M., Mistry K. S., Johnson J. C., Reid O. G. & Blackburn J. L. Probing exciton diffusion and dissociation in single-walled carbon nanotube–C60 heterojunctions. J. Phys. Chem. Lett. 7, 1794–1799 (2016). 27127916 10.1021/acs.jpclett.6b00604

[b37] Wei X., Vardeny Z. V., Sariciftci N. S. & Heeger A. J. Absorption-detected magnetic-resonance studies of photoexcitations in conjugated-polymer/C-60 composites. Phys. Rev. B 53, 2187–2190 (1996). 10.1103/physrevb.53.2187 9983706

[b38] Cook S., Katoh R. & Furube A. Ultrafast studies of charge generation in PCBM:P3HT blend films following excitation of the fullerene pcbm. J. Phys. Chem. C 113, 2547–2552 (2009).

[b39] Luponosov Y. N. . Effects of electron-withdrawing group and electron-donating core combinations on physical properties and photovoltaic performance in D-π-A star-shaped small molecules. Org. Electron. 32, 157–168 (2016).

[b40] Kozlov O. V. . Ultrafast charge generation pathways in photovoltaic blends based on novel star-shaped conjugated molecules. Adv. Energy Mater. 5, 1401657 (2015).

[b41] Dimitrov S. D. . Efficient charge photogeneration by the dissociation of PC70BM excitons in polymer/fullerene solar cells. J. Phys. Chem. Lett. 3, 140–144 (2012).

[b42] Akkuratov A. V. . Design of (X-DADAD)n type copolymers with improved optoelectronic properties for bulk heterojunction organic solar cells. Macromolecules 48, 2013–2021 (2015).

[b43] Kandada A. R. S. . Ultrafast energy transfer in ultrathin organic donor/acceptor blend. Scientific Reports 3, 2073 (2013). 23797845 10.1038/srep02073 PMC3691563

[b44] Fesser K., Bishop A. R. & Campbell D. K. Optical-absorption from polarons in a model of polyacetylene. Phys. Rev. B. 27, 4804–4825 (1983).

[b45] Österbacka R., An C. P., Jiang X. M. & Vardeny Z. V. Two-dimensional electronic excitations in self-assembled conjugated polymer nanocrystals. Science 287, 839–842 (2000). 10657294 10.1126/science.287.5454.839

[b46] Hwang I. W. . Ultrafast electron transfer and decay dynamics in a small band gap bulk heterojunction material. Adv. Mater. 19, 2307–2312 (2007).

[b47] Bakulin A. A. . Charge-transfer state dynamics following hole and electron transfer in organic photovoltaic devices. J. Phys. Chem. Lett. 4, 209–215 (2013). 26291233 10.1021/jz301883y

[b48] Shoaee S. . Charge photogeneration for a series of thiazolo-thiazole donor polymers blended with the fullerene electron acceptors PCBM and ICBA. Adv. Funct. Mater. 23, 3286–3298 (2013).

[b49] Guo J., Ohkita H., Benten H. & Ito S. Charge generation and recombination dynamics in poly(3-hexylthiophene)/fullerene blend films with different regioregularities and morphologies. J. Am. Chem. Soc. 132, 6154–6164 (2010). 20373809 10.1021/ja100302p

[b50] Grancini G. . Hot exciton dissociation in polymer solar cells. Nat Mater 12, 29–33 (2013). 23223127 10.1038/nmat3502

[b51] Bartesaghi D. & Koster L. J. A. The effect of large compositional inhomogeneities on the performance of organic solar cells: A numerical study. Adv. Funct. Mater. 25, 2013–2023 (2015).

[b52] Hoppe H. & Sariciftci N. S. Morphology of polymer/fullerene bulk heterojunction solar cells. J. Mater. Chem. 16, 45–61 (2006).

[b53] Yang X. N., van Duren J. K. J., Janssen R. A. J., Michels M. A. J. & Loos J. Morphology and thermal stability of the active layer in poly(p-phenylenevinylene)/methanofullerene plastic photovoltaic devices. Macromolecules 37, 2151–2158 (2004).

[b54] Martens T. . Disclosure of the nanostructure of MDMO-PPV:PCBM bulk hetero-junction organic solar cells by a combination of spm and tem. Synth. Met. 138, 243–247 (2003).

[b55] Schmidt-Hansberg B. . Moving through the phase diagram: Morphology formation in solution cast polymer-fullerene blend films for organic solar cells. Acs Nano 5, 8579–8590 (2011). 22004659 10.1021/nn2036279

[b56] Brady M. A., Su G. M. & Chabinyc M. L. Recent progress in the morphology of bulk heterojunction photovoltaics. Soft Matter 7, 11065–11077 (2011).

[b57] Kim J. Y. & Frisbie D. Correlation of phase behavior and charge transport in conjugated polymer/fullerene blends. J. Phys. Chem. C. 112, 17726–17736 (2008).

[b58] Shrotriya V., Ouyang J., Tseng R. J., Li G. & Yang Y. Absorption spectra modification in poly(3-hexylthiophene):methanofullerene blend thin films. Chem. Phys. Lett. 411, 138–143 (2005).

[b59] Mikhnenko O. V. . Temperature dependence of exciton diffusion in conjugated polymers. J. Phys. Chem. B 112, 11601–11604 (2008). 18729397 10.1021/jp8042363

[b60] Akselrod G. M. . Subdiffusive exciton transport in quantum dot solids. Nano Lett. 14, 3556–3562 (2014). 24807586 10.1021/nl501190s

[b61] Kohler A. & Bassler H. Electronic processes in organic semiconductors: An introduction. (Wiley-VCH Verlag GmbH & Co. KGaA, Weinheim, Germany, 2015).

[b62] Wu W.-R. . Competition between fullerene aggregation and poly(3-hexylthiophene) crystallization upon annealing of bulk heterojunction solar cells. Acs Nano 5, 6233–6243 (2011). 21749108 10.1021/nn2010816

[b63] Chen W. . Hierarchical nanomorphologies promote exciton dissociation in polymer/fullerene bulk heterojunction solar cells. Nano Lett. 11, 3707–3713 (2011). 21823620 10.1021/nl201715q

[b64] Collins B. A., Tumbleston J. R. & Ade H. Miscibility, crystallinity, and phase development in P3HT/PCBM solar cells: Toward an enlightened understanding of device morphology and stability. J. Phys. Chem. Lett. 2, 3135–3145 (2011).

[b65] Caruso D. & Troisi A. Long-range exciton dissociation in organic solar cells. Proceedings of the National Academy of Sciences 109, 13498–13502 (2012). 10.1073/pnas.1206172109 PMC3427073 22869702

[b66] Wenger O. S. How donor−bridge−acceptor energetics influence electron tunneling dynamics and their distance dependences. Acc. Chem. Res. 44, 25–35 (2011). 20945886 10.1021/ar100092v

[b67] Moses D., Dogariu A. & Heeger A. J. Ultrafast photoinduced charge generation in conjugated polymers. Chem. Phys. Lett. 316, 356–360 (2000).

[b68] Zhong C. . Ultrafast charge transfer in operating bulk heterojunction solar cells. Adv. Mater. (2015). 10.1002/adma.201405284 25677734

[b69] Kawashima K., Tamai Y., Ohkita H., Osaka I. & Takimiya K. High-efficiency polymer solar cells with small photon energy loss. Nat. Commun. 6 (2015). 10.1038/ncomms10085 PMC4686756 26626042

[b70] Min J. . Alkyl chain engineering of solution-processable star-shaped molecules for high-performance organic solar cells. Adv. Energy Mater. 4, 1301234 (2014).

[b71] Liu Y. . Solution-processed small-molecule solar cells: Breaking the 10% power conversion efficiency. Sci. Rep. 3 (2013). 10.1038/srep03356 PMC3842540 24285006

[b72] Wienk M. M. . Efficient methano[70]fullerene/MDMO-PPV bulk heterojunction photovoltaic cells. Angew. Chem. Int. Ed. 42, 3371–3375 (2003). 10.1002/anie.200351647 12888961

[b73] van Bavel S. S., Sourty E. & With G. d. & Loos, J. Controlling the 3D nanoscale organization of bulk heterojunction polymer solar cells. Chin. J. Polym. Sci. 27, 85−92 (2009). 10.1021/nl8014022 18642962

[b74] Verploegen E., Miller C. E., Schmidt K., Bao Z. & Toney M. F. Manipulating the morphology of P3HT–PCBM bulk heterojunction blends with solvent vapor annealing. Chem. Mater. 24, 3923–3931 (2012).

[b75] Cerullo G., Nisoli M., Stagira S. & De Silvestri S. Sub-8-fs pulses from an ultrabroadband optical parametric amplifier in the visible. Opt. Lett. 23, 1283 (1998). 18087499 10.1364/ol.23.001283

[b76] Yeremenko S., Baltuska A., de Haan F., Pshenichnikov M. S. & Wiersma D. A. Frequency-resolved pump-probe characterization of femtosecond infrared pulses. Opt. Lett. 27, 1171 (2002). 18026397 10.1364/ol.27.001171

[b77] Gordon R. G. Molecular collisions and depolarization of fluorescence in gases. J. Chem. Phys. 45, 1643 (1966).

[b78] Drori T., Holt J. & Vardeny Z. V. Optical studies of the charge transfer complex in polythiophene/fullerene blends for organic photovoltaic applications. Phys. Rev. B. 82, 075207 (2010).

[b79] Bakulin A. A., Martyanov D. S., Paraschuk D. Y., Pshenichnikov M. S. & van Loosdrecht P. H. M. Ultrafast charge photogeneration dynamics in ground-state charge-transfer complexes based on conjugated polymers. J. Phys. Chem. B 112, 13730–13737 (2008). 18842009 10.1021/jp8048839

[b80] Bakulin A. A., Zapunidy S. A., Pshenichnikov M. S., van Loosdrecht P. H. M. & Paraschuk D. Y. Efficient two-step photogeneration of long-lived charges in ground-state charge-transfer complexes of conjugated polymer doped with fullerene. PCCP 11, 7324–7330 (2009). 19672545 10.1039/b905249f

[b81] Treat N. D. & Chabinyc M. L. Phase separation in bulk heterojunctions of semiconducting polymers and fullerenes for photovoltaics. Annu. Rev. Phys. Chem. 65, 59–81 (2014). 24689796 10.1146/annurev-physchem-040513-103712

[b82] Feron K., Belcher W., Fell C. & Dastoor P. Organic solar cells: Understanding the role of Förster resonance energy transfer. Int. J. Mol. Sci. 13, 17019 (2012). 23235328 10.3390/ijms131217019 PMC3546737

[b83] Bartesaghi D. . Competition between recombination and extraction of free charges determines the fill factor of organic solar cells. Nat. Commun. 6, 7083 (2015). 25947637 10.1038/ncomms8083 PMC4432638

[b84] Bartesaghi D., Turbiez M. & Koster L. J. A. Charge transport and recombination in PDPP5T:[70]PCBM organic solar cells: The influence of morphology. Org. Electron. 15, 3191–3202 (2014).

[b85] Jiang Z. Gixgui, a matlab-based software for visualization and reduction of grazing incidence x-ray scattering data, http://www.aps.anl.gov/Sectors Sector8/Operations/GIXSGUI.html.

