# Supplementary material for: Bulk heterojunction morphology of polymer:fullerene blends revealed by ultrafast spectroscopy
Source: Sci Rep. 2016 Nov 8;6:36236. doi: 10.1038/srep36236 (PMC5099942; doi:10.1038/srep36236)
Supplement: Supplementary Information [file srep36236-s1.pdf]

Bulk heterojunction morphology of polymer:fullerene blends revealed by ultrafast spectroscopy

Almis Serbenta[1,†], Oleg V. Kozlov[1,2,†], Giuseppe Portale[1], Paul H.M. van Loosdrecht[1,§], and Maxim S. Pshenichnikov[1]*

[1]Zernike Institute for Advanced Materials, University of Groningen, Groningen, the Netherlands.

[2]International Laser Center and Faculty of Physics, Moscow State University, Russian Federation.

† These authors contributed equally

§ Present address: Department of Physics, University of Cologne, Cologne, Germany

*Corresponding author, e-mail: m.s.pchenitchnikov@rug.nl

Supplementary Information consists of 17 sections:

Supplementary Section 1. Optical densities and absorption coefficients 3

Supplementary Section 2. Polaron absorption 5

Supplementary Section 3. Background subtraction 5

Supplementary Section 4. Absorption spectra of RRe-P3HT blends 7

Supplementary Section 5. X-Ray measurements 7

Supplementary Section 6. Monte-Carlo modelling with the X-Ray data as an input.................. 10

Supplementary Section 7. Photoluminescence (PL) quenching 11

Supplementary Section 8. Atomic Force Microscopy 14

Supplementary Section 9. Scanning Electron Microscopy (SEM) 15

Supplementary Section 10. Transmission Electron Microscopy 16

Supplementary Section 11. Influence of energetic disorder 18

Supplementary Section 12. Hole transfer times.. 20

Supplementary Section 13. Accuracy of domain size retrieval from the Monte-Carlo simulations 22

Supplementary Section 14. Generalization of the proposed method to modern donor-acceptor systems 25

Supplementary Section 15. Excitation power dependences 25

Supplementary Section 16. Exciton kinetic parameters from time-resolved PL 26

Supplementary Section 17. PL energy shift.. 28

References 29

Supplementary Section 1. Optical densities and absorption coefficients

Supplementary Figure 1 shows the red flanks of the absorption spectra of the three pristine polymers (orange curves) and $PC_{71}BM$ (brown curves). Based on the highest contrast between $PC_{71}BM$/polymer excitations (i.e. the ratio of the absorption spectra of the $PC_{71}BM$ and the polymer, green lines), the excitation wavelength was selected as 680 nm for both RRa-P3HT and RRe-P3HT mixed with $PC_{71}BM$, and 630 nm for $PC_{71}BM$:MDMO-PPV.

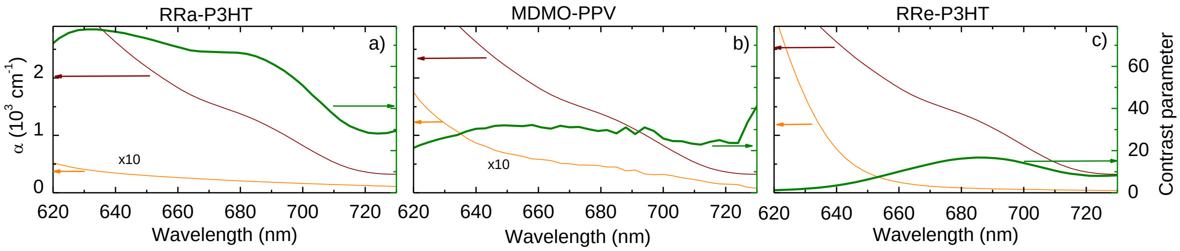

Supplementary Figure 1 Absorption coefficient of $PC_{71}BM$ (brown lines) and polymers (orange lines), and the ratio (contrast parameter) between $PC_{71}BM$ and polymer absorption (green lines) for the three polymers studied: (a) RRa-P3HT, (b) MDMO-PPV, (c) RRe-P3HT. Absorption coefficients of RRa-P3HT and MDMO-PPV are multiplied by a factor of 10.

For normalization of the transients by the number of absorbed photons the optical densities (ODs) of all samples were measured at the excitation wavelengths (Supplementary Figure 2). Apparently, ODs do not scale linearly with the $PC_{71}BM$ content which prompted us to measure film thicknesses (Supplementary Figure 3). The film thicknesses appear to vary greatly for different blend compositions because of the differences in substrate surface wetting and solution viscosity.

Next, absorption coefficients α were calculated using the following relation:

$$\alpha = \frac{OD}{L \cdot \ln(10)}, \qquad \text{(S3.1)}$$

where L is sample thickness. As expected, the resulted absorption coefficients scale linearly with the $PC_{71}BM$ content (Supplementary Figure 4).

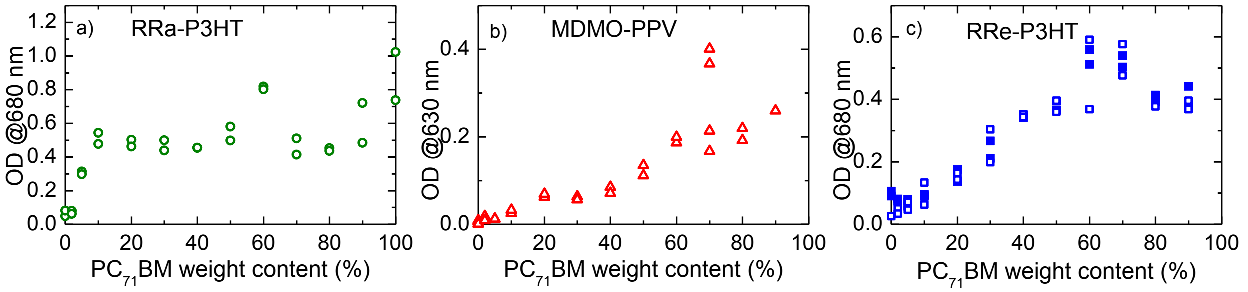

Supplementary Figure 2 Optical densities of polymer:$PC_{71}BM$ blends at the excitation wavelengths: a) RRa-P3HT at 680 nm, b) MDMO-PPV at 630 nm and c) RRe-P3HT at 680 nm. Two series of identical samples were measured with the same $PC_{71}BM$ load. Due to light scattering in the RRe-P3HT blends with low $PC_{71}BM$ content, the optical densities in (c) were measured by the absorption spectrometer (full squares) and a laser operating at 660 nm (open squares).

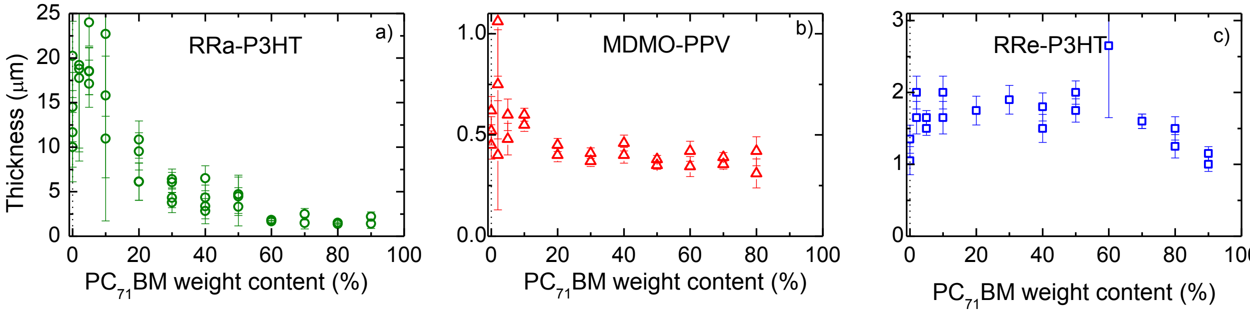

Supplementary Figure 3 Thickness of the films with various fullerene content: a) RRa-P3HT:$PC_{71}BM$, b) MDMO-PPV:$PC_{71}BM$ and c) RRe-P3HT:$PC_{71}BM$. Two series of identical samples were measured with the same $PC_{71}BM$ load. The error bars originate from irregularities of the film thickness between different samples due to the drop casting sample preparation procedure. As PIA signals are normalized by the absorption (i.e. *not* by the film thickness), the thickness uncertainty does not affect the normalized PIA signals.

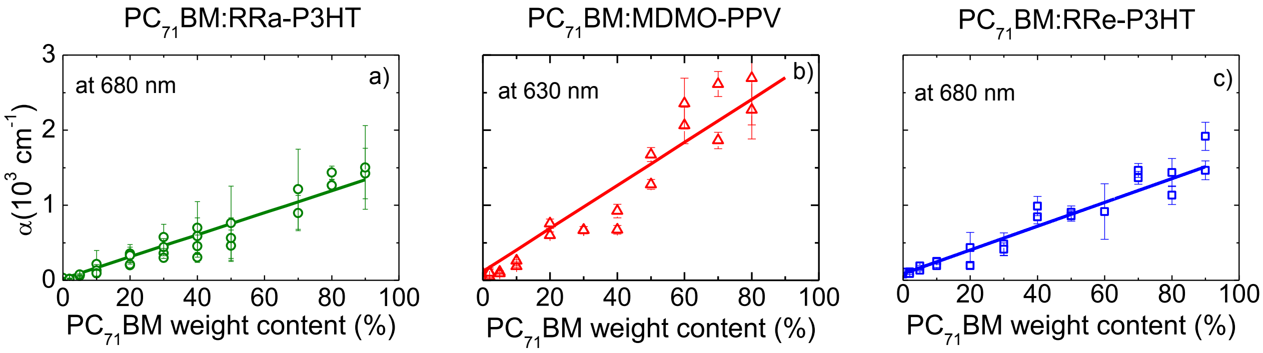

Supplementary Figure 4 Absorption coefficients (symbols) of (a) $PC_{71}BM$:RRa-P3HT, (b) $PC_{71}BM$:MDMO-PPV and (c) $PC_{71}BM$:RRe-P3HT, calculated according to Eq. S3.1 at 680 nm (a and c) and 630 nm (b). The lines are fits with the linear function to the experimental data. The difference in the slope between (a)&(c) and (b) is due to different wavelengths: at 630 nm, $PC_{71}BM$ absorbs by a factor of ~2 more than at 680 nm. Two series of identical samples were measured with the same $PC_{71}BM$ load.

Supplementary Section 2. Polaron absorption

To measure the PIA transients, the probe wavelength should be chosen close to the polaron absorption maxima. The polaron spectra for the MDMO-PPV and RRe-P3HT have been published in Refs. [1, 2] so that only the spectrum of RRa-P3HT was measured (Supplementary Figure 5). The low- and high-energy polaron peaks are located around ~0.5 eV and ~0.95 eV, respectively. The low-energy polaron peak almost does not change its position at different delays which warrant a single probe wavelength.

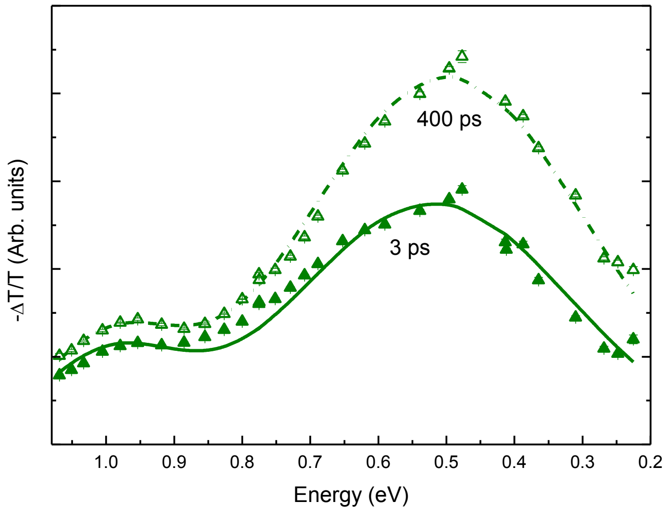

Supplementary Figure 5 Transient IR PIA spectra of RRa-P3HT:$PC_{71}BM$ 1:9 blend (green triangles) after photoexcitation at 680 nm. The closed and open symbols correspond to delays of 3 ps and 400 ps, respectively. The lines are fits with two Gaussian functions.

Supplementary Section 3. Background subtraction

Supplementary Figure 6 shows the isotropic components of the PIA signal $-\Delta T/T$ of the blends as they were measured. The PIA response of the pristine polymer in the blends (Supplementary Figure 6, black dashed lines) is negligibly low in all samples except for 10% and 20% blends with RRe-P3HT. This IR response is most probably due to CT excitons generated in RRe-P3HT[3]. Therefore, the response of pristine polymer film was scaled according to the polymer absorption fraction in the corresponding blends (0.35 and 0.2 for 10% and 20% $PC_{71}BM$ content, respectively) and directly subtracted from the corresponding transients.

The IR $PC_{71}BM$ response was identified [4] as originated from excited-state absorption of the $PC_{71}BM$ excitons. For its background subtraction, the experimental data were Monte-Carlo (MC) simulated as a sum of the true hole polaron response and the $PC_{71}BM$ exciton response. The relative cross-section of the IR $PC_{71}BM$ response was calculated as follows. We assume that

for the blends with low $PC_{71}BM$ content (10-20%), 100% exciton harvesting is achieved as no large $PC_{71}BM$ domains are formed. Therefore, the PIA signal at long delays originates solely from the hole polarons with no contribution of the $PC_{71}BM$ response. The direct IR $PC_{71}BM$ exciton response was measured in the neat $PC_{71}BM$ film (Supplementary Figure 6, the bottom panel). Its relative cross-section was calculated as a ratio of the IR $PC_{71}BM$ exciton response to the hole polaron response, to amount to 0.3, 0.1 and <0.05 for RRa-P3HT, MDMO-PPV and RRe-P3HT, respectively.

For blends with each polymer, 100% efficiency of charge separation was assumed in the sample with maximal amplitude amongst all transients (note that the amplitudes in blends for different polymers cannot be compared directly due to the different polaron absorption cross-sections). Then each transient in the set of the particular polymer was normalized to the amplitude obtained. After this normalization, the signal amplitude directly provides the relative charge yield, i.e. the exciton harvesting efficiency.

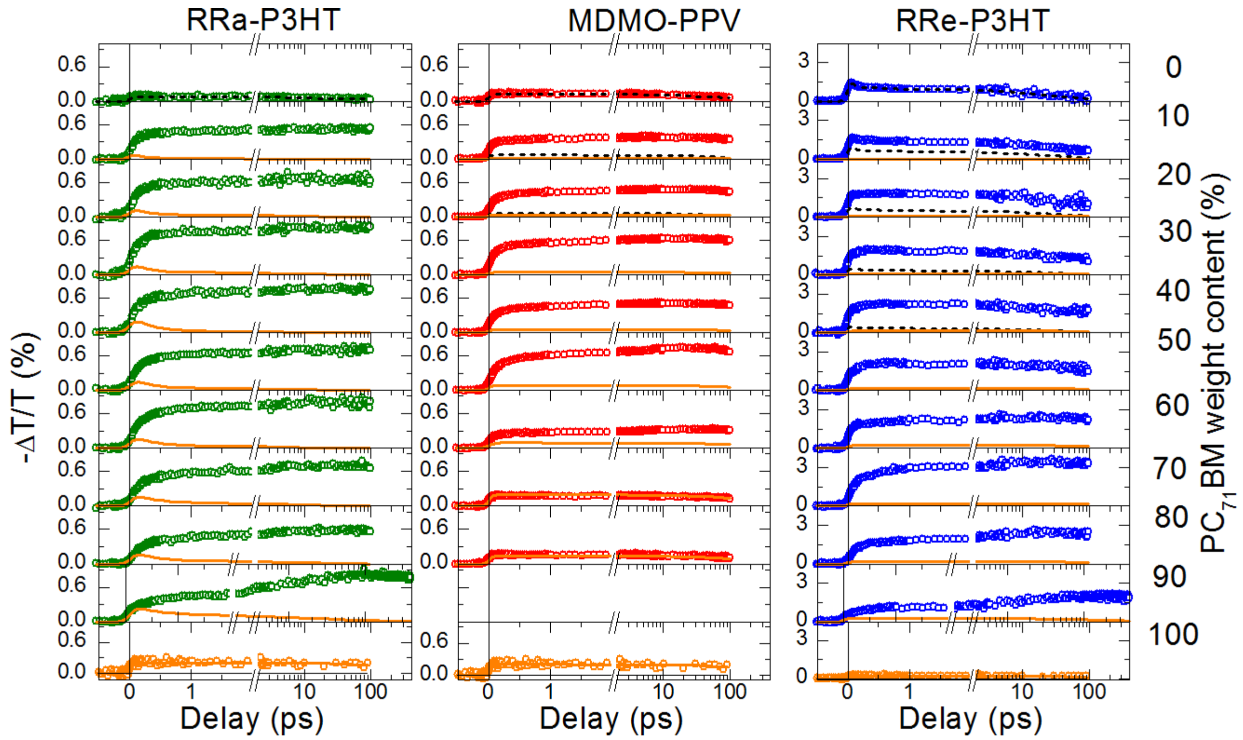

Supplementary Figure 6 PIA transients as obtained in the experiment, for different weight ratios of $PC_{71}BM$ to polymer: (a) RRa-P3HT, (b) MDMO-PPV, and (c) RRe-P3HT. Symbols represent the experimental points. The solid orange lines represent the $PC_{71}BM$ contribution as obtained from the MC simulations, while the dashed black lines are the pristine polymer contribution. The 90% MDMO-PPV sample produced similar to neat $PC_{71}BM$ response and therefore is not shown.

Supplementary Section 4. Absorption spectra of RRe-P3HT blends

Supplementary Figure 7 shows the red flank of absorption spectra of RRe-P3HT blends. The disappearance of RRe-P3HT absorption shoulder at 620 nm due to the broken crystallinity [5, 6] when the $PC_{71}BM$ content changes from 60% to 70% is observable.

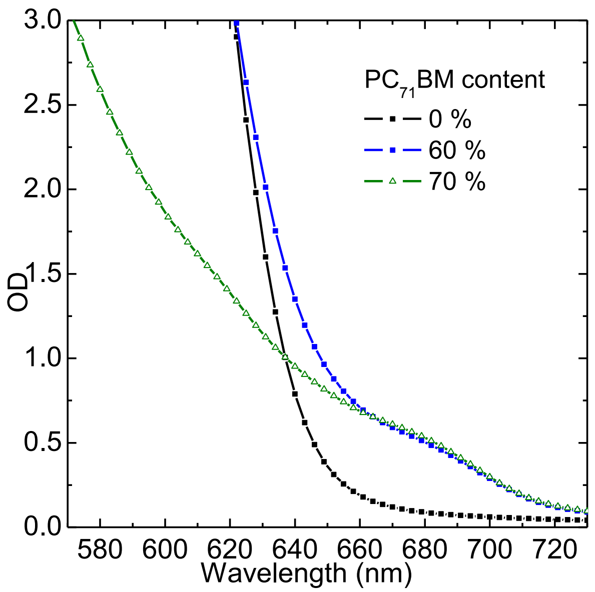

Supplementary Figure 7 Linear absorption of RRe-P3HT:$PC_{71}BM$ blends with different fullerene content (indicated). Note the disappearance of RRe-P3HT absorption shoulder at ~620 nm when $PC_{71}BM$ load changes from 60% to 70%.

Supplementary Section 5. X-Ray measurements

Morphology of RRe-P3HT-based samples with $PC_{71}BM$ loadings of >40% was independently verified by grazing-incidence small-angle X-ray scattering (GISAXS) and grazing-incidence wide-angle X-ray scattering (GIWAXS) measurements. The results are summarized in Supplementary Figure 8 and Supplementary Table 1.

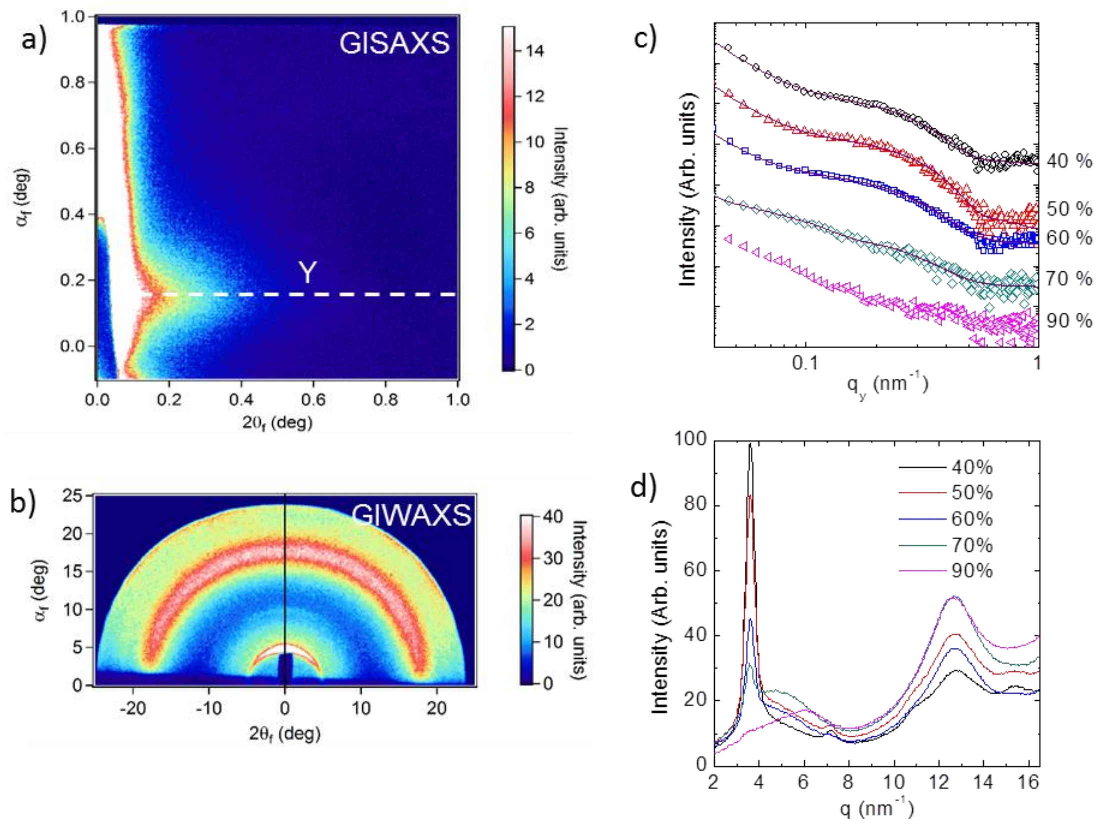

Supplementary Figure 8 GISAXS (a) and GIWAXS (b) images for the RRe-P3HT-based film with 60% $PC_{71}BM$ content. Curves in panel (c) are the in-plane GISAXS intensity cuts taken at the Yoneda peak position (Y). The curves are shifted vertically for clarity. The open symbols show experimental data while the solid lines are best model fits (see below). (d) Total integrated GIWAXS intensities.

Supplementary Table 1 Summary of the RRe-P3HT:$PC_{71}BM$ film internal structures extracted from the GISAXS and GIWAXS analysis.

$PC_{71}BM$ content (%)	Mixed phase size 1 (nm)	Mixed phase size 2 (nm)	Mixed phase size 3 (nm)	Large domain size (nm)
40	1.8	15	-	>100
50	1.9	15	-	
60	1.9	14.5	-	
70	2.1	14	23.8	
90	2.1	-	-	

Fitting of the GISAXS in-plane intensities in the framework of the distorted wave-born approximation was performed with the FitGISAXS software package [7]. A model composed by spherical objects polydisperse in size describes well the intensity in the range q_y = 0.1–1 nm^{-1} and it is generally accepted in literature to describe GISAXS scattering in P3HT:$PC_{71}BM$ films

[8]. All the samples except the one with 70% $PC_{71}BM$ content can be modeled by one population of spheres. To fit the data for the 70% $PC_{71}BM$ sample, two populations of spheres have been considered. The polydispersity in the sphere diameter was about 0.2 for all the samples. The intensity upturn visible for q_y values lower than 0.1 nm^{-1} is generated by the existence of large $PC_{71}BM$ domains [9]. This upturn is generally modeled by a Debye−Anderson−Brumberger (DAB) equation with correlation length, ξ, for the characterization of the domains [10]. For all our samples, the correlation length is larger than the maximum length scale detected in our measurements (d>100 nm). The size is in agreement with the TEM results (Supplementary Table 2), different preparation methods of the samples notwithstanding.

Both the P3HT crystalline content and the fraction of small 2 nm $PC_{71}BM$ domains show a linear behavior with increasing the $PC_{71}BM$ content up to 60% (Supplementary Figure 9). Above 70%, a strong deviation from the linearity occurs. This deviation is assigned to the broken crystallinity of P3HT domains and formation of amorphous P3HT phase (see also Supplementary Figure 7), in accordance with the literature [5, 6]. For the $PC_{71}BM$ 2 nm fraction, a slight deviation from linearity occurs for blends with >60% $PC_{71}BM$ content, which indicates gradual formation of large $PC_{71}BM$ domains (be that 15 nm or >100 nm in size). For the 90% $PC_{71}BM$ blend, the fraction of 2 nm domains drop significantly indicating sufficient amount of the large domains formed, which is in line with dramatic decrease of PIA signal (Figure 2c in the Main Text).

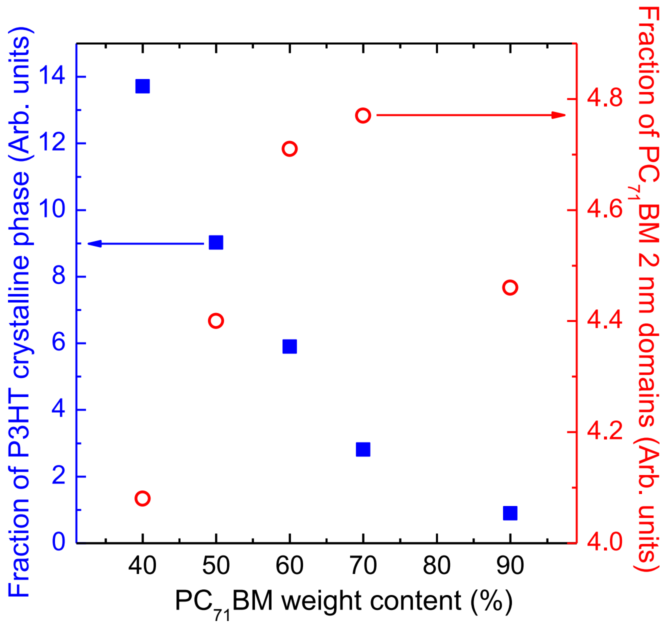

Supplementary Figure 9 Relative fractions of the P3HT crystalline phase (black) and of the $PC_{71}BM$ 2 nm domain phase (red) as a function of the $PC_{71}BM$ content in the blend.

Supplementary Section 6. Monte-Carlo modelling with the X-Ray data as an input

In the Main Text we model the mixed phase as small $PC_{71}BM$ domain with a single size spheres. According to the X-ray measurements, for the RRe-P3HT:$PC_{71}BM$ blends the mixed phase consists of domains with characteristic sizes of 2 nm and 15 nm with different shares (Supplementary Table 1, Supplementary Figure 9). In this case, the output of the simulations reflects the effective domain size which lies between 2 and 15 nm and depends on the participation ratio of the two domains (Figure 3c in the Main Text). We performed the MC simulations with mixed phase modelled as two spherical domains of 3 and 15 nm with different shares as the X-ray data suggest (3 nm size instead of 2 nm was chosen due to the limitation of MC simulations). The simulations perfectly describe the transients and show increasing of the fraction of 15 nm domains for higher $PC_{71}BM$ concentration (Supplementary Figure 10). As the exact choice of the model does not affect the dynamics nor the exciton harvesting efficiency, it does not play any role for the solar cell operation.

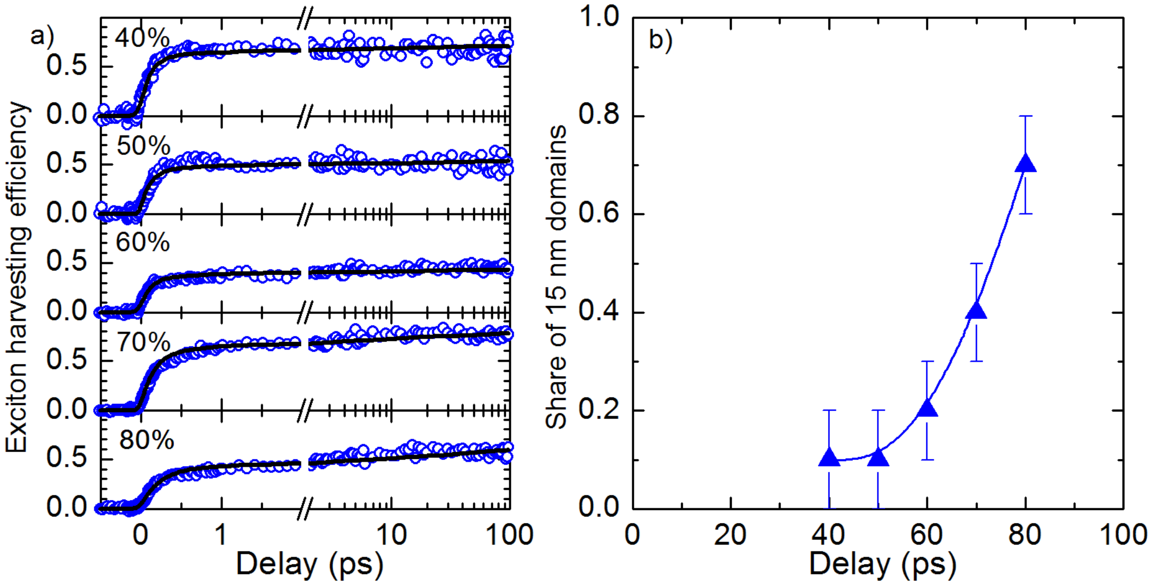

Supplementary Figure 10 (a) MC simulations for RRe-P3HT:$PC_{71}BM$ blends with different $PC_{71}BM$ content (indicated) with mixed phase modelled as 3 nm and 15 nm spheres. The open symbols represent experimental data while the solid lines show simulated transients. (b) Share of 15 nm domains in the mixed phase as resulted from the MC modelling.

Supplementary Section 7. Photoluminescence (PL) quenching

The results of PIA measurements were independently verified by the photoluminescence (PL) quenching measurements. For each sample, time-resolved PL transients were measured by a Hamamatsu C5680 streak-camera system after 650 nm excitation, with time resolution of ~10 ps. The color glass longpass filter RG695 was placed before the polychromator to filter the stray excitation light. Representative time-integrated spectra of the samples based on three different polymers are shown in Supplementary Figure 11. Generally, the spectra of the BHJ blends consist of three contributions: $PC_{71}BM$ PL, polymer PL, and PL of the charge-transfer (CT) state[11, 12].

In RRa-P3HT-based blends, red flanks of PL spectra of RRa-P3HT and $PC_{71}BM$ are very close (Supplementary Figure 11a). Besides, CT-state PL develops already at low $PC_{71}BM$ concentrations. However, high contrast of $PC_{71}BM$ excitation (Supplementary Figure 1) and PL spectral filtering in the 680-710 region allow for extraction of the PL signal that is mainly assigned to $PC_{71}BM$ PL (Supplementary Figure 12a. The transients clearly show that PL is strongly quenched, down to the width of the apparatus function at all $PC_{71}BM$ concentrations

except 90% where slight elongation of the PL transient is observed. These results are in line with those derived from PIA (Figure 3 of the main text) where strong quenching is predicted because of fine intermixing of RRa-P3HT and $PC_{71}BM$ in the blends. Nonetheless, due to aforementioned reasons PL experiments provide no ground for deriving any qualitative information on the $PC_{71}BM$ domain size.

MDMO-PPV based blends suffer from the complications similar to the RRa-P3HT case (Supplementary Figure 12b). However, because the $PC_{71}BM$ domains are much larger, time-resolved PL does provide some useful information (Supplementary Figure 12b). PL transients demonstrate increased PL quenching as $PC_{71}BM$ content decreases, in full correspondence to Figure 3 of the main text. Both PIA and PL quenching methods demonstrate similar exciton harvesting efficiencies (Supplementary Figure 11) which proves the validity of the PIA measurements. Note that the initial amplitude of the transients decreases at lower $PC_{71}BM$ concentrations because the time-resolved PL measurements cannot catch PL originating from small $PC_{71}BM$ domains due to limited time resolution (~ 10 ps). This is in high contrast with the PIA measurements (Figure 2 of the main text) where all the times from 0.1 to 100 ps are readily captured.

Finally, for the RRe-P3HT-based blends, the PL spectra are also strongly contaminated by the polymer emission (Supplementary Figure 11c, green lines). However, due to low contrast of $PC_{71}BM$ excitation, low $PC_{71}BM$ PL quantum yield (QY) but high PL QY of RRe-P3HT, it is virtually impossible to deconvolute $PC_{71}BM$ PL from the total PL. At blends with <60% $PC_{71}BM$ concentration, the PL is mainly originates from P3HT crystals (note increase of PL amplitude for those blends in Supplementary Figure 12c). As a result, PL of the large $PC_{71}BM$ domains can barely be seen only at 90% $PC_{71}BM$ concentration. This excludes deriving any quantitative information of the $PC_{71}BM$ domain size, in contrast with the PIA measurements (see Figure 3 of the main text).

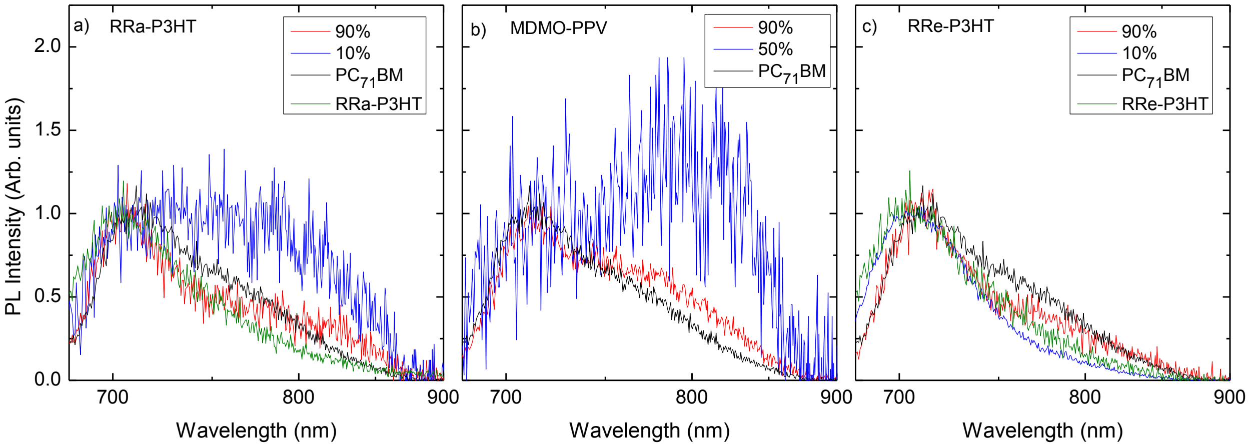

Supplementary Figure 11 Representative PL spectra after 650 nm excitation for (a) RRa-P3HT, (b) MDMO-PPV and (c) RRe-P3HT based blends with different $PC_{71}BM$ concentrations (indicated). The spectra are normalized to $PC_{71}BM$ maximum at 710 nm. Blue flanks of the spectra are cut off by a long-pass RG695 filter placed before the polychromator to filter out the excitation light.

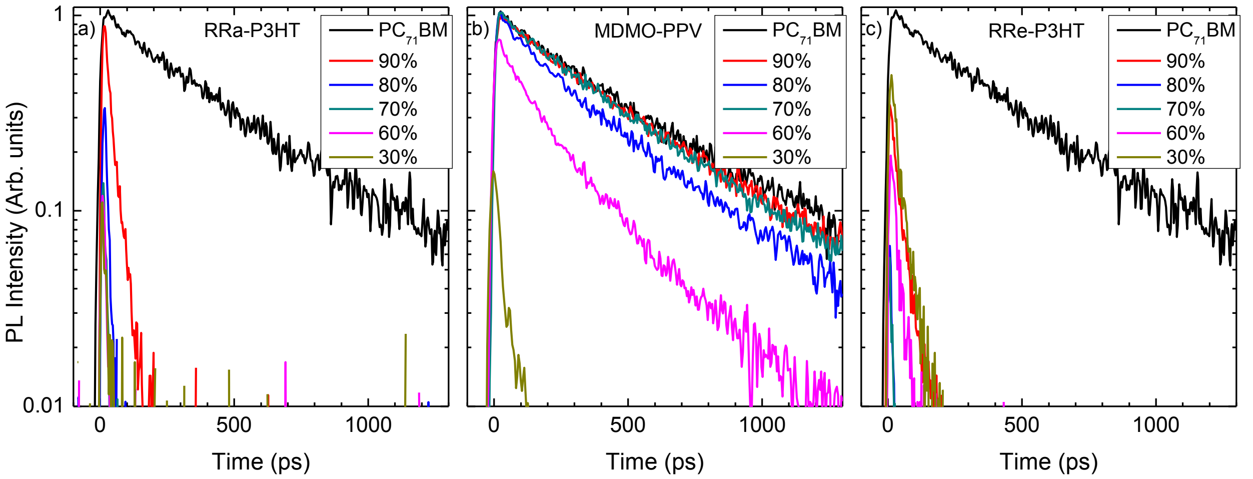

Supplementary Figure 12 PL transients for (a) RRa-P3HT, (b) MDMO-PPV and (c) RRe-P3HT based blends with $PC_{71}BM$ after 650 nm excitation. Concentrations of $PC_{71}BM$ are indicated in the legend. Transients are integrated in the 680-710 (a,c) or 680-730 (b) nm spectral ranges and normalized by the blend absorption at the wavelength of excitation.

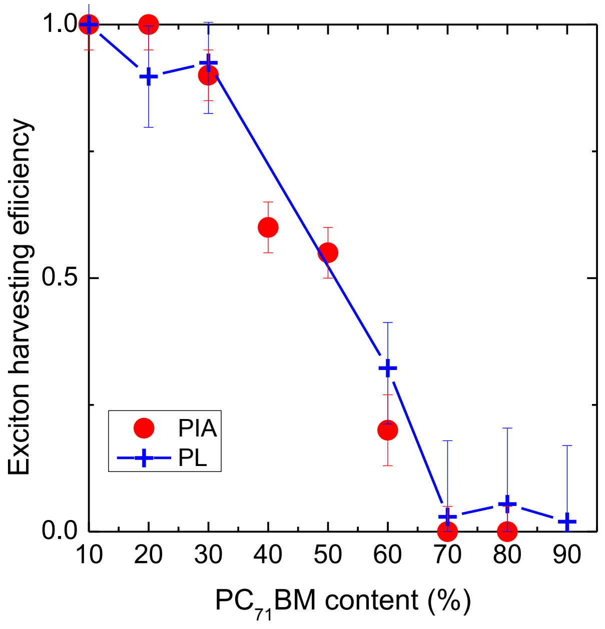

Supplementary Figure 13 Exciton harvesting efficiency within first 100 ps as obtained from PIA (dots) and PL (crosses/line) measurements.

Supplementary Section 8. Atomic Force Microscopy

Supplementary Figure 14a shows images of the surface roughness obtained from AFM in the tapping mode for the very same MDMO-PPV: $PC_{71}BM$ samples that were used for PIA and PL measurements. The images were used to estimate characteristic $PC_{71}BM$ domain size by calculating two-dimensional autocorrelation function (2D-AC) using the Gwyddion software (Supplementary Figure 14b). The central parts of the 2D-AC functions were fitted with the 2D Gaussian which widths were averaged to obtain the characteristic size of $PC_{71}BM$ domains (Fig. 4b, Main Text).

For the P3HT based blends, it is known that surface morphology does not represent the bulk morphology due to severe vertical phase segregation [13]. In full accord with this, AFM was performed on the P3HT-based samples but shown no contrast.

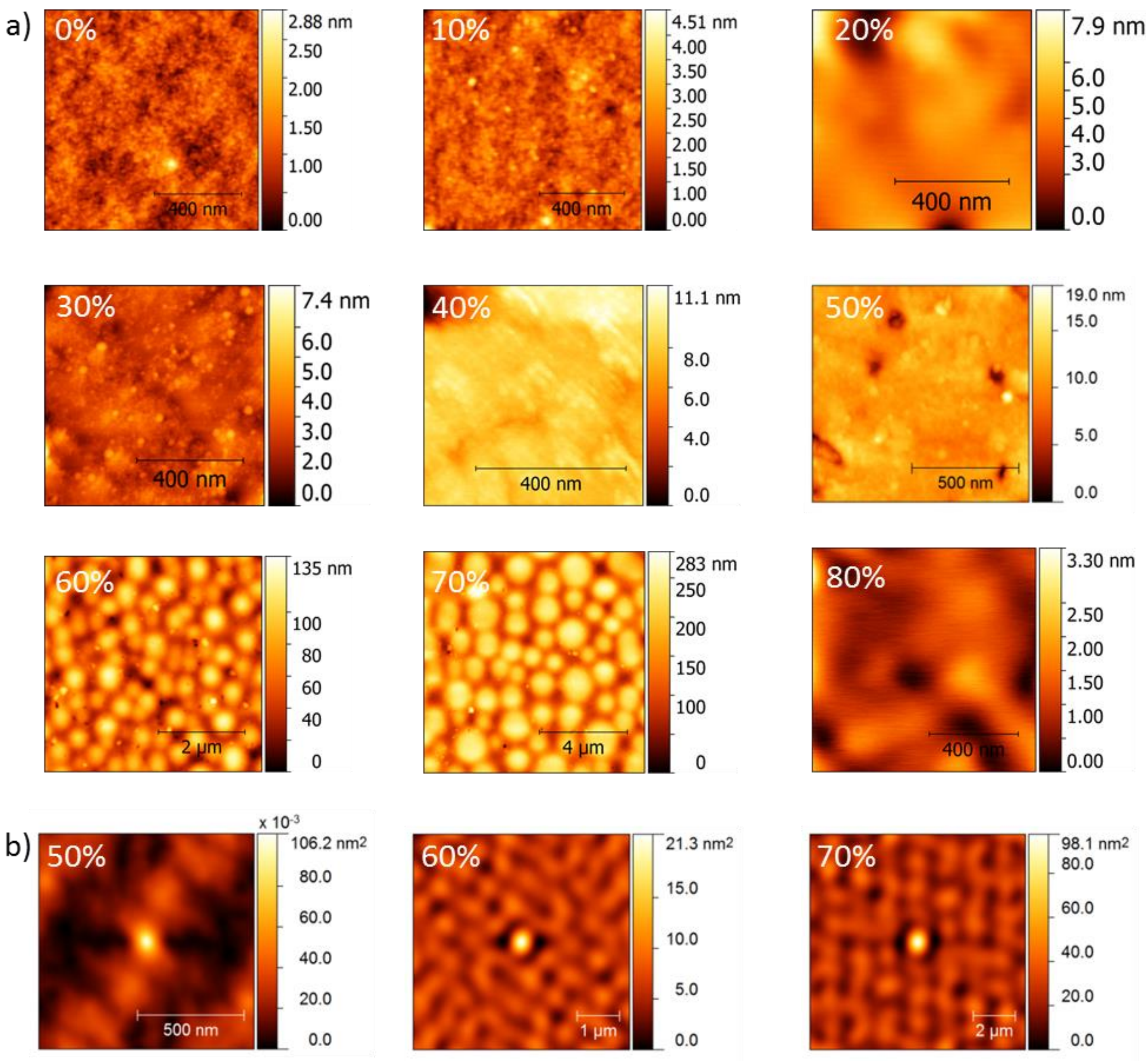

Supplementary Figure 14 (a) AFM images (in tapping mode) of surface roughness for MDMO-PPV:$PC_{71}BM$: blends with different $PC_{71}BM$ weight ratios (indicated). The bright colored spherical shapes in 60% and 70% images are the $PC_{71}BM$ domains. (b) show 2D autocorrelation functions for 50-70% AFM images.

Supplementary Section 9. Scanning Electron Microscopy (SEM)

Supplementary Figure 15 shows an SEM image of MDMO-PPV:$PC_{71}BM$ sample with 70% $PC_{71}BM$ load obtained by Philips XL30S SEM FEG. The very same sample was used for the SEM as for the PIA measurements. Before the SEM measurements, the sample surface was covered by a thin sputtered layer of gold to reduce sample degradation (due to charging effect) and enhance the signal to noise ratio. The size of $PC_{71}BM$ domains is comparable or larger than the film thickness, similarly to the other report [14]. Therefore, the surface topography measured

by AFM actually represents the bulk size of $PC_{71}BM$ domains. The RRe-P3HT-based samples did not reveal any resolvable features which could be assigned to the $PC_{71}BM$ domains and therefore the results are not shown.

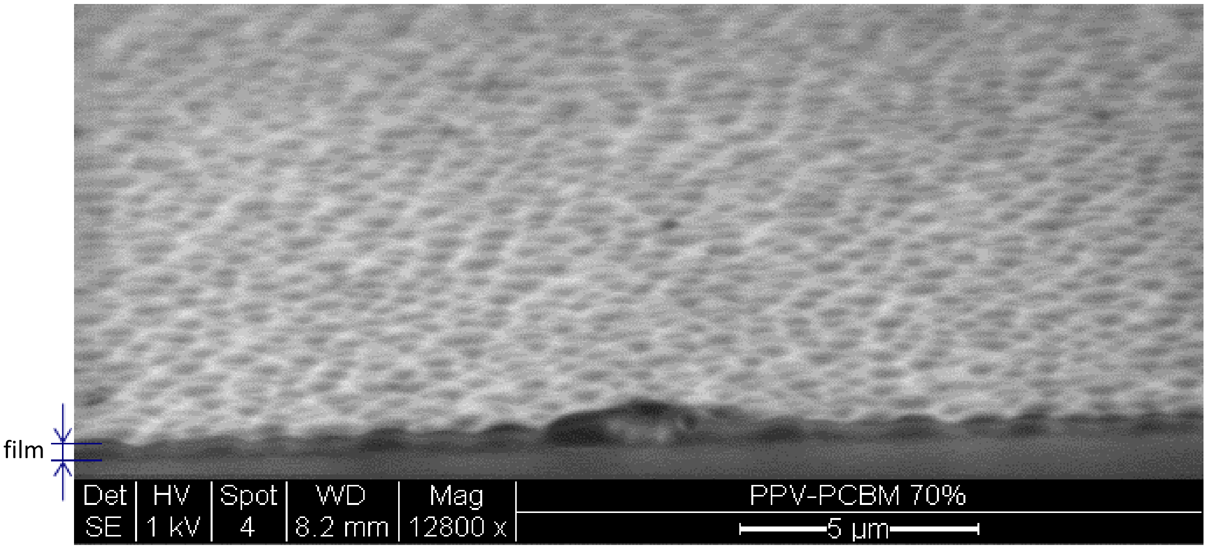

Supplementary Figure 15 SEM image of MDMO-PPV:$PC_{71}BM$ film with 70% $PC_{71}BM$ load. Due to the tilt of the sample both the cross-sectional view and the tilted-surface topography are visible. The film thickness is shown at the left.

Supplementary Section 10. Transmission Electron Microscopy

For the P3HT-based blends AFM is not able to provide any valuable morphology information because of i) relatively small $PC_{71}BM$ domain size, and ii) presence of P3HT-rich layer on top of the casted film which affects the blend topology [13]. Therefore, the size of large $PC_{71}BM$ domains in the RRe-P3HT blends was measured using Transmission Electron Microscopy (TEM). For the TEM measurements, free-standing thin films were prepared in a clean room using the procedures described in Refs. [15, 16]. RRe-P3HT and $PC_{71}BM$ were dissolved separately in ortho-dichlorobenzene at concentrations of 5 g/L and mixed with appropriate volumes to obtain $PC_{71}BM$ contents of 50%, 70% and 90%. Glass substrates were consequently i). cleaned with soap and demineralized water, ii.) sonicated in demineralized water, iii). sonicated in acetone, iv). sonicated in isopropanol, v). sonicated in demineralized water, vi). dried on the centrifuge, vii). exposed to UV light. The PEDOT:PSS solution in water was spin-coated on the cleaned glass substrates at 1000 rounds per minute (RPM) for 1 min and then baked in the oven at 100°C for 20 min to dry. Mixed solutions of $PC_{71}BM$:RRe-P3HT were

spin coated at 2000 RPM with the lid closed for 5 seconds and then at 1000 RPM for 1 min with the open lid.

The prepared thin films were sliced into small rectangles (approximately ~3x3 mm) and immersed into demineralized water in order to dissolve the PEDOT:PSS layer and lift the films from the substrate. The 400 mesh copper grids were used to pick up the freestanding films from water and put into Philips CM120 electron microscope operating at 120 keV. Just before performing the TEM measurement, all $PC_{71}BM$:RRe-P3HT films were stained with iodine vapors for several minutes, a procedure known to improve the contrast for $PC_{71}BM$:polymer blends [17]. The iodine vapors were obtained by dissolving solid iodine into 99.5% purity methanol. The films of $PC_{71}BM$:RRe-P3HT on copper grids were kept above the iodine solution for a few minutes for staining.

Supplementary Figure 16-15 show the TEM images of RRe-P3HT:$PC_{71}BM$ blends with $PC_{71}BM$/polymer weight content 50%, 70%, and 90% of $PC_{71}BM$. With an increase of $PC_{71}BM$ content, the bright features become more pronounced and abundant suggesting that the bright features are related to the higher density of $PC_{71}BM$ molecules. The sizes of $PC_{71}BM$domains were estimated by averaging the sizes of 20 arbitrary chosen bright regions for each RRe-P3HT:$PC_{71}BM$ blend. The resulted domain sizes are summarized in Supplementary Table 2.

Supplementary Table 2 Estimated $PC_{71}BM$ domain sizes

$PC_{71}BM$ content (%)	Estimated $PC_{71}BM$ domain size (nm)
50	90±15
70	110±25
90	125±25

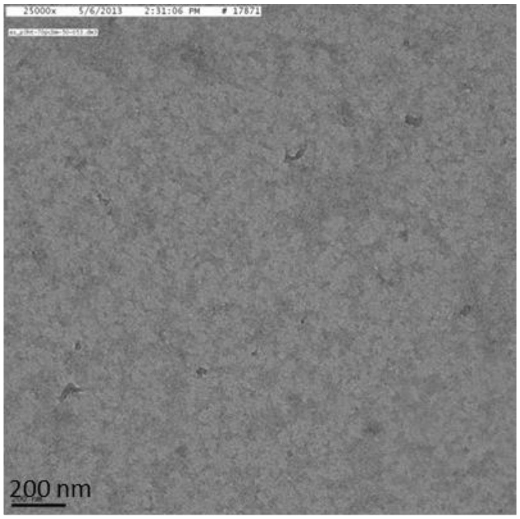

Supplementary Figure 16 Transmission electron microscope image of the $PC_{71}BM$:RRe-P3HT film with the $PC_{71}BM$ content of 50%. Brighter and darker areas are $PC_{71}BM$ and RRe-P3HT enriched, respectively.

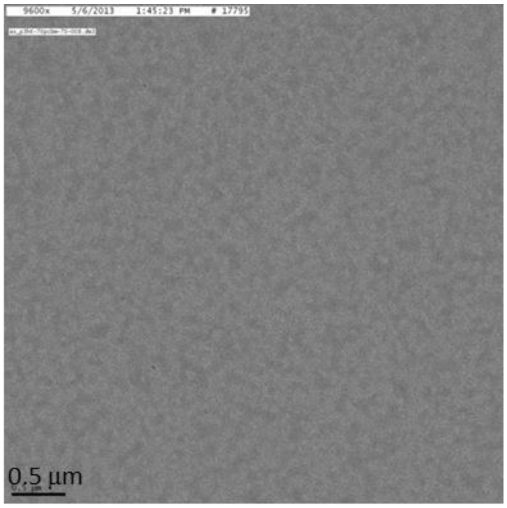

Supplementary Figure 17 Transmission electron microscope image of the $PC_{71}BM$:RRe-P3HT film with the $PC_{71}BM$ content of 70%. Brighter and darker areas are $PC_{71}BM$ and RRe-P3HT enriched, respectively.

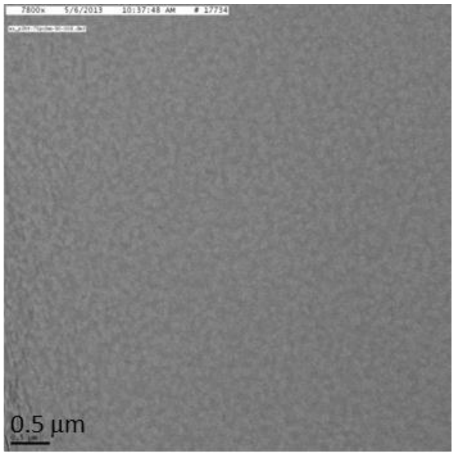

Supplementary Figure 18 Transmission electron microscope image of the $PC_{71}BM$:RRe-P3HT film with the $PC_{71}BM$ content of 90%. Brighter and darker areas are $PC_{71}BM$ and RRe-P3HT enriched, respectively.

Supplementary Section 11. Influence of energetic disorder

The energetic disorder has a great influence on the exciton dynamics as it creates low-energy trap states which significantly slow exciton diffusion. Moreover, the presence of neighboring sites with different energies makes the probability of hopping to different directions unequal thereby effectively reducing the dimensionality of the diffusion process. As a reminder, the difference between diffusion coefficient in 3D and 1D is $\sqrt{3}$.

Supplementary Figure 19 shows representative trajectories of the excitons with infinite lifetime in media with and without disorder obtained from the MC simulations (parameters of the

simulations are given in the Main Text). Without energetic disorder, the diffusion process is uniform in time with each subsequent step bringing the exciton to the new site. In the disordered medium, plateaus are formed on the trajectories where the exciton spends more time in the low-energy sites from where it is hard to escape. This leads to shorter displacements and a wide distribution of the average hopping times (Supplementary Figure 20) in contrast to the no-disorder case where the exciton spends identical times on each site.

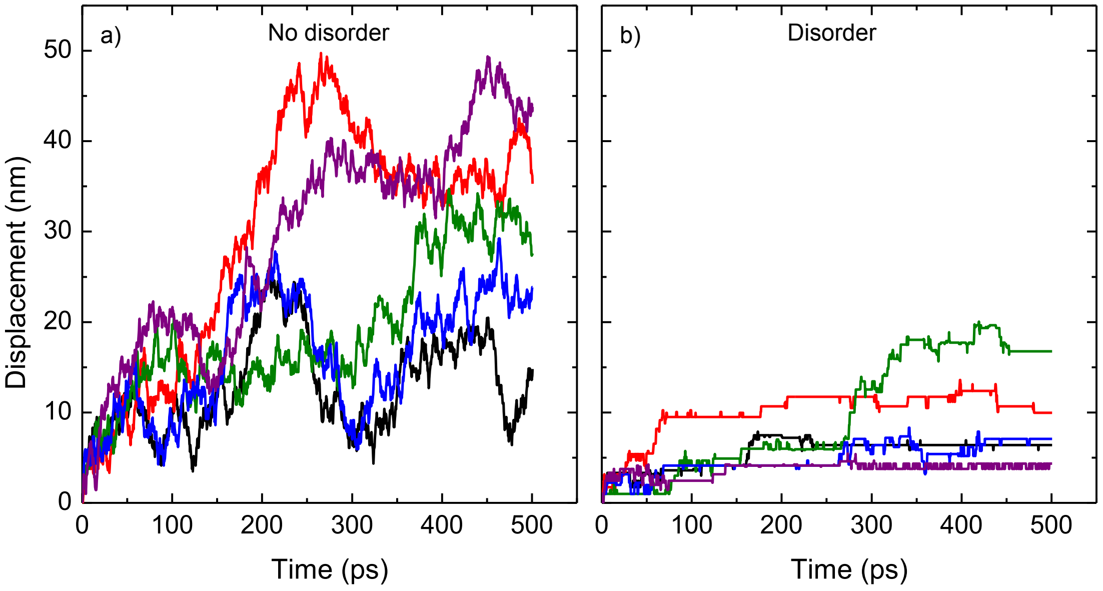

Supplementary Figure 19 Simulated displacement of 5 different excitons without (a) and with (b) energetic disorder. Initial diffusion coefficient is $D=4.5\cdot 10^{-3}$ cm^2/s, energetic disorder in (b) is σ=70 meV and the room temperature kT=26 meV.

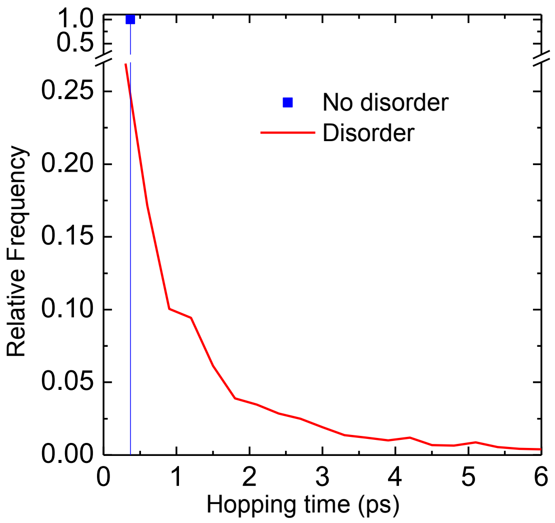

Supplementary Figure 20 Distribution of average exciton hopping times with (red line) and without (blue dot) energetic disorder. Without the disorder, the distribution is a δ-function.

Supplementary Section 12. Hole transfer times

To reproduce the short-time part of the experimental data, the finite hole transfer (HT) time was set as free parameter for each sample in the MC simulations. The resulted dependence of the HT time on the $PC_{71}BM$ content is shown in Supplementary Figure 21. The HT time gradually decreases from ~0.1 ps to ~0.5 ps with the increase of $PC_{71}BM$ concentration (and hence the domain size). We attribute this behavior to the long-range hole transfer [18-20] from next to the outer layer of $PC_{71}BM$ domains, which probability increases with increasing of the fullerene domain size (see Main Text). However, the effect of exciton screening in the large fullerene domains and/or exciton delocalization cannot be ruled out as influencing the HT time, too. We emphasize that the ultrafast component of HT time in the 30-50 fs range [21, 22] was clearly observed in these experiments but instead of treated separately was included in the apparatus function in the MC simulations for the sake of simplicity.

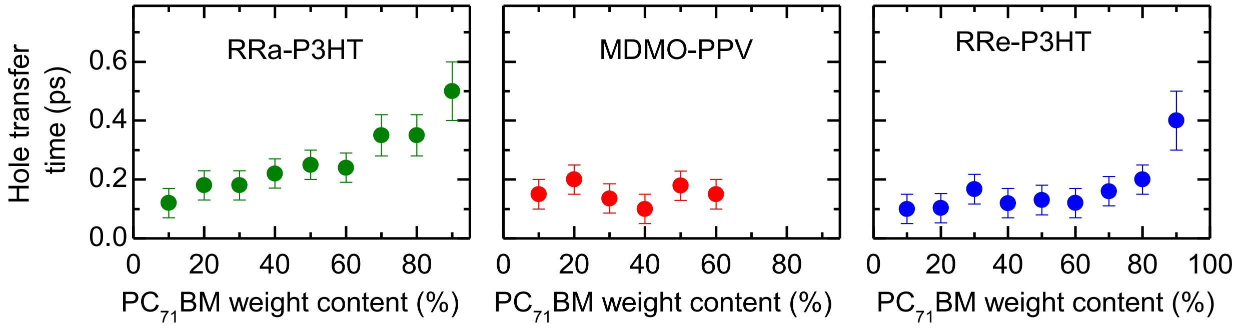

Supplementary Figure 21 HT time as obtained from MC simulations

In the context of hole transfer times, we comment briefly on the report by Kandada *et al.*[23]. In this paper, the authors claim that in the layered organic donor-acceptor blend of RRe-P3HT and PCBM "hole transfer from PCBM to the polymer takes place on the 100 ps time scale". Taken literally, this statement might be read as the hole transfer time amounts to 100 ps. However, it should be pointed out that according to Kandada *et al.*, the PCBM excitons are created mainly due to Dexter energy transfer after RRe-P3HT excitation, and therefore the PCBM exciton density follows the e^{-r/r_0} dependence, where r_0 is the Dexter energy transfer distance of ~1 nm. This means that there would be a spatial distribution of the excitons in the PCBM-rich domains (see e.g. Fig.2 of Ref [23]) which implies that PCBM exciton diffusion takes place prior PCBM exciton splitting into the charges at the interface. This scenario does not seem to have been contemplated by Kandada *et al*. who considered diffusion of the P3HT excitons only.

We performed MC simulations on this scenario for a bilayer RRe-P3HT/PCBM structure (Supplementary Figure 22), the outcome of which excellently explains the growing in time polaron production (red curve in Fig.3d of [23]) without invoking 100-ps hole transfer time but taking into consideration PCBM exciton diffusion. The actual hole transfer time was most probably not resolved in Ref. [23] due to the competing Dexter energy transfer process that occurs at the similar timescale of a hundred of fs.

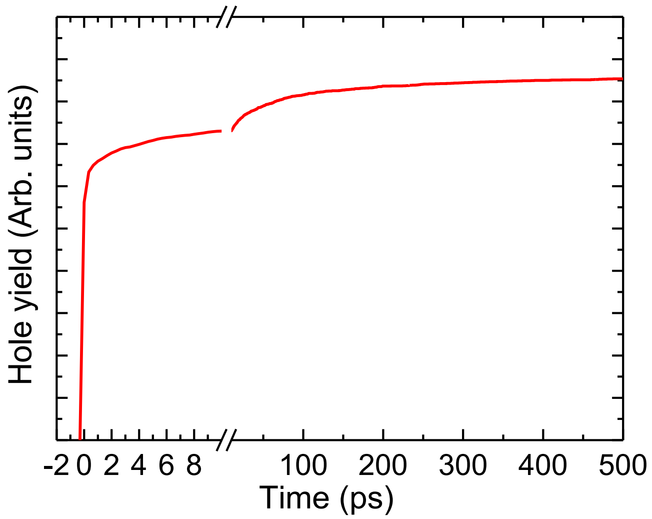

Supplementary Figure 22 Calculated hole yield in a bilayer structure populated by Dexter energy transfer mechanism. The Dexter energy transfer was assumed to occur from the whole 5-nm layer of P3HT according to e^{-r/r_0} dependence; all other parameters are identical to those used in MC simulations of Fig. 2 in the main text.

Supplementary Section 13. Accuracy of domain size retrieval from the Monte-Carlo simulations

Kinetic parameters of the MC simulations, i.e. energetic disorder, exciton lifetime and the hopping time were derived from time-resolved PL on pristine films of $PC_{71}BM$ (Supplementary Section 12) and used for retrieval of the $PC_{71}BM$ domain size (Fig. 3, Main Text). However, one can argue that these parameters may in turn depend on the $PC_{71}BM$ domain size, with the data derived from the pristine films being a limiting case of a very big $PC_{71}BM$ domain. As a consequence, the problem of size retrieval loses self-consistency and as such might lack a unique solution for the mixed polymer:$PC_{71}BM$ phase. Here we show, by examining the stability of the MC simulations with respect to those parameters, that this is not the case. In particular, we demonstrate the retrieved sizes do not change upon vast variations of the kinetic parameters thereby lifting the requirement of their precise determination for each particular blend (which would have been hardly feasible).

Supplementary Figure 23 shows the MC simulations output for two mixed phase domain sizes: 6 nm (RRa-P3HT:$PC_{71}BM$ with 10% $PC_{71}BM$ concentration) and 8 nm (RRe-P3HT:$PC_{71}BM$ with 80% $PC_{71}BM$ concentration) taken as the representative cases, with the aforementioned parameters varied in a wide range. As follows from the figure, the hopping time

has very moderate influence on the dynamics. Only when the hopping time is increased by a factor of 3, the MC simulations begin to fail to reproduce the early-time experimental data for $PC_{71}BM$ concentration of 80%. This is due to the fact that the early-time part of the PIA transient is mainly determined by the excitons arrived from the interfacial and the next to the interfacial layers, i.e. when the exciton cooling has not occurred yet. In all other cases, the variations of the hopping time as much as a factor of 3 do not result in any appreciable deterioration of the fit quality, considering experimental noise.

Due to a similar reason, the energy disorder also has very little effect on the simulated curves. From the mixed phase, the excitons are mainly extracted before equilibration of the diffusion coefficient D (Supplementary Figure 24) and, therefore, the variations in energy disorder do not have any serious influence on harvesting efficiency from the mixed phase. Perhaps, some effect could be observed in the dynamics for relatively large domains (>8 nm) but here the degree of freedom in hopping time is strongly confined by the fact that the 8-nm domains represent bulk material and therefore the values derived from PL quenching experiments, should be applied.

Finally, the exciton lifetime does not affect the early-time dynamics either because of the extraction of excitons from the mixed phase occurs in time that is much shorter than the exciton lifetime. The concern about the exciton lifetime is especially valid because the $PC_{71}BM$ lifetime changes, albeit very moderately - from 900 ps for non-interacting $PC_{71}BM$ molecules dissolved in a solid matrix of PMMA to 650 ps in the pristine $PC_{71}BM$ film (Supplementary Figure 27). This reduction most probably results from PL self-quenching due to intermolecular interactions in the film of $PC_{71}BM$. Nonetheless, the dynamics for the mixed phase do not change with the exciton lifetime variation within this limit (Supplementary Figure 23).

Therefore, we conclude that MC simulations are stable for domain sizes retrieval for both mixed phase and large domains with respect to the kinetic parameters that arguably may depend on the $PC_{71}BM$ domain size. This is attributed to extremely fast extraction of the excitons from the $PC_{71}BM$ domains. On the other hand, the large $PC_{71}BM$ domains behave as the bulk material so that the parameters derived from the PL measurements can be safely used.

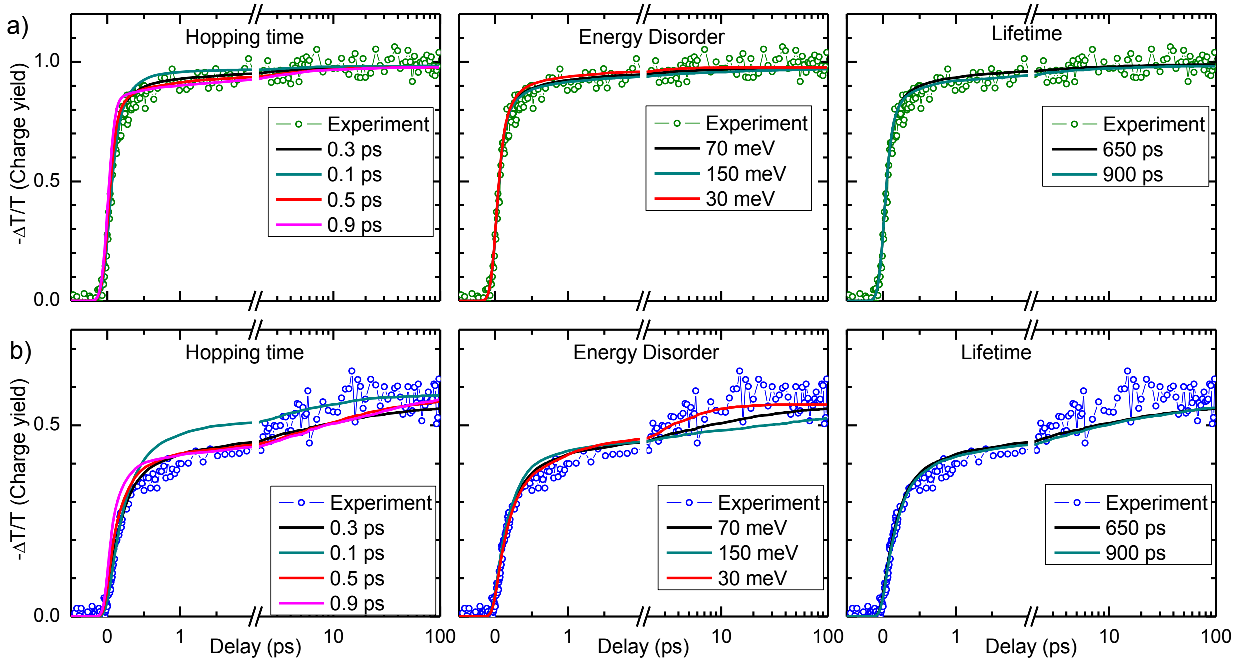

Supplementary Figure 23 MC simulations for (a) RRa-P3HT:$PC_{71}BM$ with 10% $PC_{71}BM$ concentration (retrieved mixed phase domain size 6 nm) and (b) RRe-P3HT:$PC_{71}BM$ with 80% $PC_{71}BM$ concentration (retrieved mixed phase domain size 8 nm). The parameters of the MC simulations (hopping time τ, energy disorder σ and exciton lifetime T_1) were varied in wide range (indicated in the legend). The values deduced from the PL quenching measurements are τ=0.3 ps , σ=70 meV and T_1=650 ps.

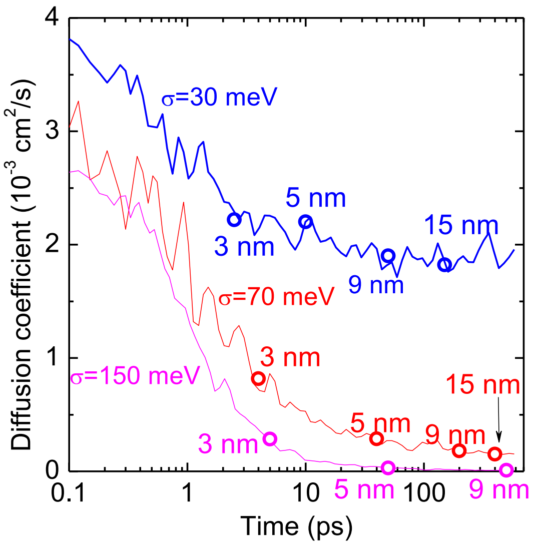

Supplementary Figure 24 Simulated exciton diffusion coefficient dependence on time for energy disorder of σ=70 meV (red line; the value deduced from the PL quenching measurements), σ=150 meV (magenta line) and σ=30 meV (blue line). Open circles indicate the domain size from which the excitons are extracted at the given time.

Supplementary Section 14. Generalization of the proposed method to modern donor-acceptor systems

To demonstrate the applicability of the proposed technique to the modern donor-acceptor systems, we performed the analysis of photovoltaic blend based on a novel star-shaped small molecule N(Ph-2T-DCV-Me)$_3$ [24] with 85% PC$_{71}$BM content. The excitation wavelength was set to 550 nm; therefore, both donor and PC$_{71}$BM are excited approximately equally. As excitons from N(Ph-2T-DCV-Me)$_3$ phase and interfacial PC$_{71}$BM excitons dissociate within <200 fs [24], the respective PIA contributions can be considered as a step-like function at the timescale of PC$_{71}$BM exciton diffusion. By including this response into the MC simulations, we readily separate the N(Ph-2T-DCV-Me)$_3$ and diffusion-delayed PC$_{71}$BM contributions. Using the same kinetic parameters for PC$_{71}$BM as in the Main Text, the PC$_{71}$BM domain size is retrieved as 8±1 nm. This clearly demonstrates that the high contrast between PC$_{71}$BM and polymer/small molecule excitations is a convenient but not essential requirement.

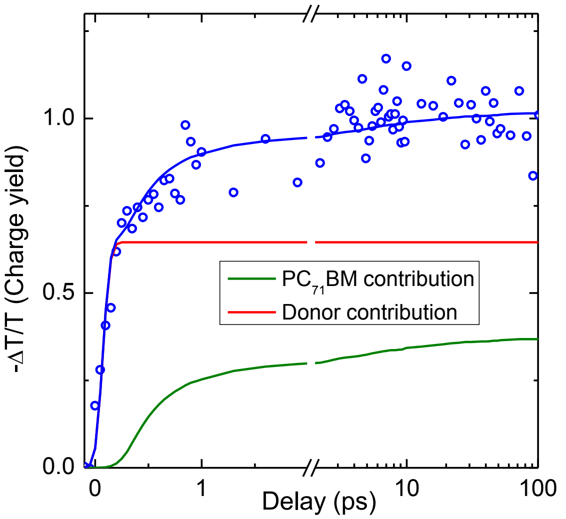

Supplementary Figure 25 Measured (dots) and simulated (blue line) PIA transients for N(Ph-2T-DCV-Me)$_3$: PC$_{71}$BM 1:5 blend. N(Ph-2T-DCV-Me)$_3$ (via electron transfer) and PC$_{71}$BM (via hole transfer) contributions to the total PIA signal are shown in red and green, respectively. For details on the system, refer to Ref. [24].

Supplementary Section 15. Excitation power dependences

To ensure the absence of undesirable effects caused by high power of excitation (such as bi-exciton annihilation, two photon absorption, higher-order nonlinearities etc.) dependences of the PIA signals on the excitation power were measured for different PC$_{71}$BM concentrations. The

representative dependences for1:1 polymer:$PC_{71}BM$ blends are shown in Supplementary Figure 26. The effects described above would have led to the nonlinear dependence (saturation) of the PIA signal on the excitation power; none were observed. The excitation power was chosen in the linear region of the PIA response of 75 μJ/cm^2 for the P3HT blends and 120 μJ/cm^2 for the MDMO-PPV blends which ensures the exciton density of lower than 10^{-3} photons/nm^3.

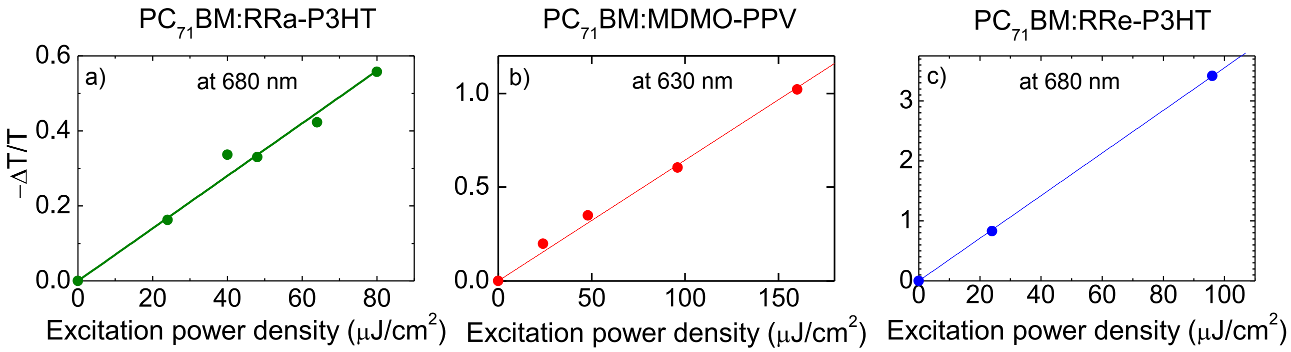

Supplementary Figure 26 Dependences of the PIA responses on excitation power density for 1:1 polymer:PCBM blends for a) RRa-P3HT, b) MDMO-PPV and c) RRe-P3HT. The excitation wavelengths are indicated in each panel. The pump-probe delay time was set at 100 ps; similar dependences were observed at delay of 3 ps.

Supplementary Section 16. Exciton kinetic parameters from time-resolved PL

The MC simulations require several parameters for the data modeling: i). $PC_{71}BM$ exciton lifetime; ii). energy disorder of excitons on different $PC_{71}BM$ molecules (which enters as the energy differences E_i - E_j in Eq. 3.1 of the main text); and iii). initial exciton hopping rate. All these parameters were obtained from independent measurements of PL quenching efficiency. $PC_{71}BM$ films mixed with TPTPA [25] quencher were manufactured under controlled TPTPA molar fractions of 50%, 25%, 12.8%, 3.2%, 0.8%, 0.1%, 0.0125%, and 0% (neat $PC_{71}BM$ film). The materials were separately dissolved in ODCB at concentrations of 20 g/l, and then mixed to achieve given molar concentrations of TPTPA.

To examine the effect of intermolecular interactions, we also measured PL of the well-separated $PC_{71}BM$ molecules diluted in a PMMA matrix. For this, PMMA was dissolved in ODCB at concentrations of 150 g/l and then mixed with $PC_{71}BM$ solution to achieve the relative concentration of ~1 $PC_{71}BM$ molecule per 2000 PMMA monomers (the averaged inter$PC_{71}BM$ distance of ~7 nm). The samples were prepared by drop-casting of the resulted solutions.

For each sample, time-resolved PL transients were measured by a Hamamatsu C5680 streak-camera system after 560 nm excitation, with time resolution of ~10 ps. To obtain the PL dynamics, the red flank of $PC_{71}BM$ PL was integrated in the spectral region of 780-850 nm, which is the least affected by $PC_{71}BM$ exciton spectral dynamics (see below).

The resulted PL decay transients were globally fit with effective exciton lifetime T_1, hopping time τ and energy disorder σ as the global fit parameters, i.e. identical for all samples (Supplementary Figure 27). The MC model was similar to that described above with exception of the grid size used. Here, the $PC_{71}BM$ film was modelled as a grid of 400x400x400 nods with periodic boundary conditions (i.e. if the exciton crosses the border it appears at the other side). TPTPA quenchers were placed randomly in the grid with the given molar fraction. At time zero, the pre-defined number of excitons (6400) was placed randomly in the $PC_{71}BM$ molecules in the grid. Once the exciton hops to the TPTPA quencher, it splits into the charges and is excluded from the simulations. As the output, the number of as-yet survived excitons is used.

PL dynamics at all concentrations were successfully simulated with a single set of parameters of T_1=650 ps, τ=0.3 ps and σ=70 meV; the obtained values are in line with those reported in the literature [26-28]. This suggests that the kinetic parameters obtained from the PL quenching measurements can be used to model the $PC_{71}BM$ domains of any size since low quencher concentrations represent bulk $PC_{71}BM$ while high quencher concentrations (50%) correspond dispersed $PC_{71}BM$ molecules in a TPTPA matrix. The fact that a single set of kinetic parameters is needed to describe as high range of quencher concentrations as 0.0125-50%, signifies similar exciton diffusion in $PC_{71}BM$ domains of different sizes.

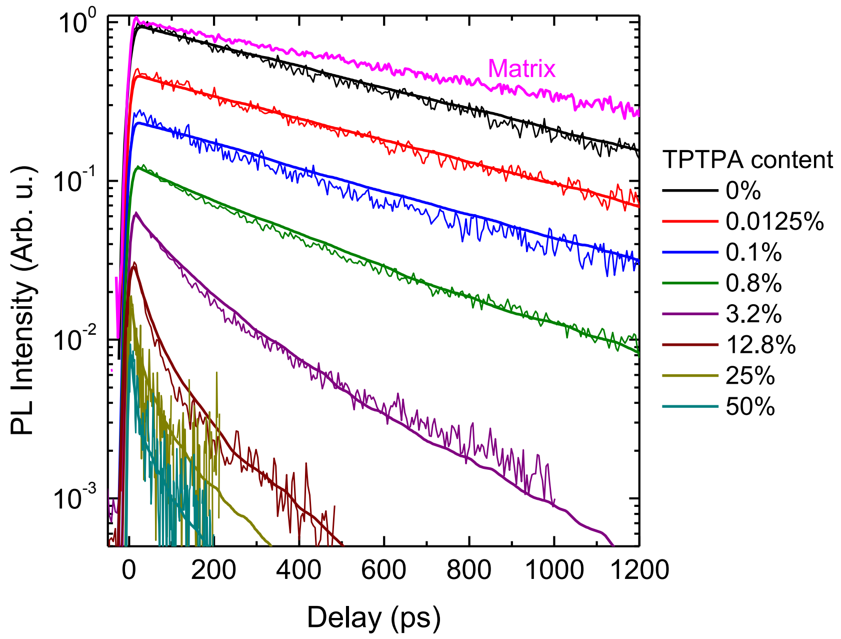

Supplementary Figure 27 Experimental $PC_{71}BM$ PL transients (thin lines) and results of the MC simulations (thick lines). The PL transient for $PC_{71}BM$ molecules strongly diluted in the PMMA matrix is shown for comparison.

Supplementary Section 17. PL energy shift

One of the crucial parameters in the MC simulations, the energy disorder of 70 meV, was independently verified by direct comparison of the PL red-shift in the neat $PC_{71}BM$ film with results of the simulations (Supplementary Figure 28a). The PL red shift is caused by exciton downhill hopping in the disordered medium [29, 30], which results in dynamical decrease of exciton mobility (Fig. 5b, Main Text). The fact that the energy relaxation is due to the downhill exciton migration is confirmed by the absence of a noticeable PL red-shift for the $PC_{71}BM$ molecules diluted in PMMA matrix, where no exciton migration occurs because of absence of intermolecular interactions (Supplementary Figure 28b, red line).

To track the energy dynamics from the MC simulation, the mean energy of the whole exciton ensemble was calculated on each simulation step. As in the simulations the density of states is centered at 0 eV, the simulated values were shifted by 1.78 eV to match the experimental conditions. Experimental and simulated energy dynamics are compared in Supplementary Figure 28b. Levelling-off of the experimental curve at short delays is due to

limited streak-camera resolution of ~10 ps. The simulated energy shift matches the experiment perfectly both in amplitude and dynamics, which confirms the validity of disorder value quoted above.

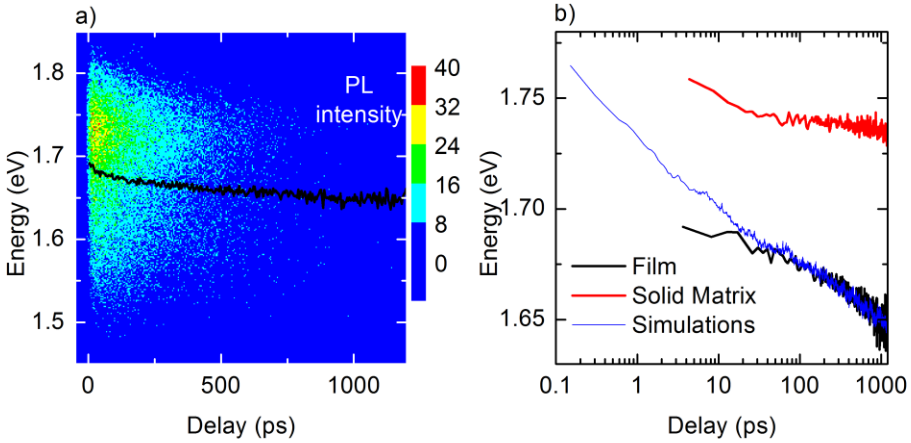

Supplementary Figure 28 (a) PL map for the neat $PC_{71}BM$ film. The mean energy value at each time is shown by the black line. The mean energy does not coincide with the PL maximum due to an asymmetric shape of the $PC_{71}BM$ PL spectrum. (b) Measured in film (black line) and PMMA matrix (red line) and simulated (blue line) energy dynamics of the PL maximum. The simulated curve is shifted by 1.78 eV to match the experimental conditions.

References

1. Österbacka, R., C.P. An, X.M. Jiang, and Z.V. Vardeny, *Two-Dimensional Electronic Excitations in Self-Assembled Conjugated Polymer Nanocrystals.* Science, 2000. **287**(5454): p. 839-842.
2. Bakulin, A.A., D.S. Martyanov, D.Y. Paraschuk, M.S. Pshenichnikov, and P.H.M. van Loosdrecht, *Ultrafast Charge Photogeneration Dynamics in Ground-State Charge-Transfer Complexes Based on Conjugated Polymers.* Journal of Physical Chemistry B, 2008. **112**(44): p. 13730-13737.
3. Drori, T., J. Holt, and Z.V. Vardeny, *Optical studies of the charge transfer complex in polythiophene/fullerene blends for organic photovoltaic applications.* Phys. Rev. B., 2010. **82**(7): p. 075207.
4. Kozlov, O.V., F. de Haan, R.A. Kerner, B.P. Rand, D. Cheyns, and M.S. Pshenichnikov, *Real-Time Tracking of Singlet Exciton Diffusion in Organic Semiconductors.* Physical Review Letters, 2016. **116**(5): p. 057402.
5. Kim, J.Y. and D. Frisbie, *Correlation of Phase Behavior and Charge Transport in Conjugated Polymer/Fullerene Blends.* J. Phys. Chem. C., 2008. **112**(45): p. 17726-17736.
6. Shrotriya, V., J. Ouyang, R.J. Tseng, G. Li, and Y. Yang, *Absorption spectra modification in poly(3-hexylthiophene): methanofullerene blend thin films.* Chem. Phys. Lett., 2005. **411**(1-3): p. 138-143.

7. Babonneau, D., *FitGISAXS: software package for modelling and analysis of GISAXS data using IGOR Pro.* Journal of Applied Crystallography, 2010. **43**(4): p. 929-936.
8. Wu, W.-R., U.S. Jeng, C.-J. Su, K.-H. Wei, M.-S. Su, M.-Y. Chiu, C.-Y. Chen, W.-B. Su, C.-H. Su, and A.-C. Su, *Competition between Fullerene Aggregation and Poly(3-hexylthiophene) Crystallization upon Annealing of Bulk Heterojunction Solar Cells.* ACS Nano, 2011. **5**(8): p. 6233-6243.
9. Wang, W., S. Guo, E.M. Herzig, K. Sarkar, M. Schindler, D. Magerl, M. Philipp, J. Perlich, and P. Muller-Buschbaum, *Investigation of morphological degradation of P3HT:PCBM bulk heterojunction films exposed to long-term host solvent vapor.* Journal of Materials Chemistry A, 2016. **4**(10): p. 3743-3753.
10. Chen, C.-Y., C.-S. Tsao, Y.-C. Huang, H.-W. Liu, W.-Y. Chiu, C.-M. Chuang, U.S. Jeng, C.-J. Su, W.-R. Wu, W.-F. Su, and L. Wang, *Mechanism and control of the structural evolution of a polymer solar cell from a bulk heterojunction to a thermally unstable hierarchical structure.* Nanoscale, 2013. **5**(16): p. 7629-7638.
11. Manca, M., C. Piliego, E. Wang, M.R. Andersson, A. Mura, and M.A. Loi, *Tracing charge transfer states in polymer:fullerene bulk-heterojunctions.* Journal of Materials Chemistry A, 2013. **1**(25): p. 7321-7325.
12. Baran, D., N. Li, A.-C. Breton, A. Osvet, T. Ameri, M. Leclerc, and C.J. Brabec, *Qualitative Analysis of Bulk-Heterojunction Solar Cells without Device Fabrication: An Elegant and Contactless Method.* Journal of the American Chemical Society, 2014. **136**(31): p. 10949-10955.
13. Vaynzof, Y., D. Kabra, L. Zhao, L.L. Chua, U. Steiner, and R.H. Friend, *Surface-Directed Spinodal Decomposition in Poly[3-hexylthiophene] and C61-Butyric Acid Methyl Ester Blends.* ACS Nano, 2011. **5**(1): p. 329-336.
14. Hoppe, H., M. Niggemann, C. Winder, J. Kraut, R. Hiesgen, A. Hinsch, D. Meissner, and N.S. Sariciftci, *Nanoscale morphology of conjugated polymer/fullerene-based bulk-heterojunction solar cells.* Adv. Funct. Mater., 2004. **14**(10): p. 1005-1011.
15. Bartesaghi, D., I.d.C. Perez, J. Kniepert, S. Roland, M. Turbiez, D. Neher, and L.J.A. Koster, *Competition between recombination and extraction of free charges determines the fill factor of organic solar cells.* Nat. Commun., 2015. **6**: p. 7083.
16. Bartesaghi, D., M. Turbiez, and L.J.A. Koster, *Charge transport and recombination in PDPP5T:[70]PCBM organic solar cells: The influence of morphology.* Org. Electron., 2014. **15**(11): p. 3191–3202.
17. Dimitrov, S.D., C.B. Nielsen, S. Shoaee, P.S. Tuladhar, J.P. Du, I. McCulloch, and J.R. Durrant, *Efficient Charge Photogeneration by the Dissociation of PC70BM Excitons in Polymer/Fullerene Solar Cells.* J. Phys. Chem. Lett., 2012. **3**(1): p. 140-144.
18. Caruso, D. and A. Troisi, *Long-range exciton dissociation in organic solar cells.* Proceedings of the National Academy of Sciences, 2012. **109**(34): p. 13498-13502.
19. Wenger, O.S., *How Donor−Bridge−Acceptor Energetics Influence Electron Tunneling Dynamics and Their Distance Dependences.* Accounts of Chemical Research, 2011. **44**(1): p. 25-35.
20. Paddon-Row, M.N., *Investigating long-range electron-transfer processes with rigid, covalently linked donor-(norbornylogous bridge)-acceptor systems.* Accounts of Chemical Research, 1994. **27**(1): p. 18-25.

21. Bakulin, A.A., J.C. Hummelen, M.S. Pshenichnikov, and P.H.M. van Loosdrecht, *Ultrafast Hole-Transfer Dynamics in Polymer/PCBM Bulk Heterojunctions.* Advanced Functional Materials, 2010. **20**(10): p. 1653-1660.
22. Serbenta, A., *Organic donor-acceptor systems: Charge generation and morphology*, 2016, University of Groningen: Groningen, The Netherlands.
23. Kandada, A.R.S., G. Grancini, A. Petrozza, S. Perissinotto, D. Fazzi, S.S.K. Raavi, and G. Lanzani, *Ultrafast Energy Transfer in Ultrathin Organic Donor/Acceptor Blend.* Scientific Reports, 2013. **3**: p. 2073.
24. Kozlov, O., Y. Luponosov, S. Ponomarenko, D. Paraschuk, N. Kausch-Busies, and M. Pshenichnikov, *Ultrafast intramolecular dynamics in novel star-shaped molecules for photovoltaic applications*, in *Ultrafast Dynamics in Molecules, Nanostructures and Interfaces*, G.G. Gurzadyan, et al., Editors. 2014, World Scientific Publishing Co. Pte. Ltd.: Singapore. p. 169-183.
25. Fischer, F.S.U., D. Trefz, J. Back, N. Kayunkid, B. Tornow, S. Albrecht, K.G. Yager, G. Singh, A. Karim, D. Neher, M. Brinkmann, and S. Ludwigs, *Highly Crystalline Films of PCPDTBT with Branched Side Chains by Solvent Vapor Crystallization: Influence on Opto-Electronic Properties.* Adv. Mater., 2014. **27**: p. 1223-1228.
26. Dimitrov, S.D., Z.G. Huang, F. Deledalle, C.B. Nielsen, B.C. Schroeder, R.S. Ashraf, S. Shoaee, I. McCulloch, and J.R. Durrant, *Towards optimisation of photocurrent from fullerene excitons in organic solar cells.* Energ. Environ. Sci., 2014. **7**(3): p. 1037-1043.
27. Fedorova, A., M.N. Berberan-Santosa, J.-P. Lefèvreb, and B. Valeur, *Picosecond time-resolved and steady-state studies of the polarization of the fluorescence of C60 and C70.* Chem. Phys. Lett., 1997. **267**(5-6): p. 467–471.
28. Amarasinghe Vithanage, D., A. Devizis, V. Abramavicius, Y. Infahsaeng, D. Abramavicius, R.C. MacKenzie, P.E. Keivanidis, A. Yartsev, D. Hertel, J. Nelson, V. Sundstrom, and V. Gulbinas, *Visualizing charge separation in bulk heterojunction organic solar cells.* Nat. Commun., 2013. **4**: p. 2334.
29. Kagan, C.R., C.B. Murray, and M.G. Bawendi, *Long-range resonance transfer of electronic excitations in close-packed CdSe quantum-dot solids.* Physical Review B, 1996. **54**(12): p. 8633-8643.
30. Crooker, S.A., J.A. Hollingsworth, S. Tretiak, and V.I. Klimov, *Spectrally Resolved Dynamics of Energy Transfer in Quantum-Dot Assemblies: Towards Engineered Energy Flows in Artificial Materials.* Physical Review Letters, 2002. **89**(18): p. 186802.
